# Flexible Non-Enzymatic Glucose Sensors: One-Step Green Synthesis of NiO Nanoporous Films via an Electro-Exploding Wire Technique

**DOI:** 10.1021/acsami.4c13653

**Published:** 2024-11-12

**Authors:** Nadeem Ahamad, Soumallya Banerjee, Chia-Chun Wei, Kuan-Cheng Lu, Akhil Pradiprao Khedulkar, Wen-Bin Jian, Sadiq Mahmood, Chih-Wei Chu, Hong-Cheu Lin

**Affiliations:** † Department of Materials Science and Engineering, National Yang Ming Chiao Tung University, Hsinchu 300093, Taiwan; ‡ Department of Electrophysics, National Yang-Ming Chiao Tung University, Hsinchu 300093, Taiwan; § International College of Semiconductor Technology, National Yang Ming Chiao Tung University, Hsinchu 300093, Taiwan; ∥ Research Center for Applied Sciences, Academia Sinica, Taipei 11529, Taiwan; ⊥ Center for Emergent Functional Matter Science, National Yang Ming Chiao Tung University, Hsinchu 300093, Taiwan

**Keywords:** nonenzymatic glucose sensor, limit of detection (LOD), NiO nanoparticles, electrochemical interactions, flexible electrode

## Abstract

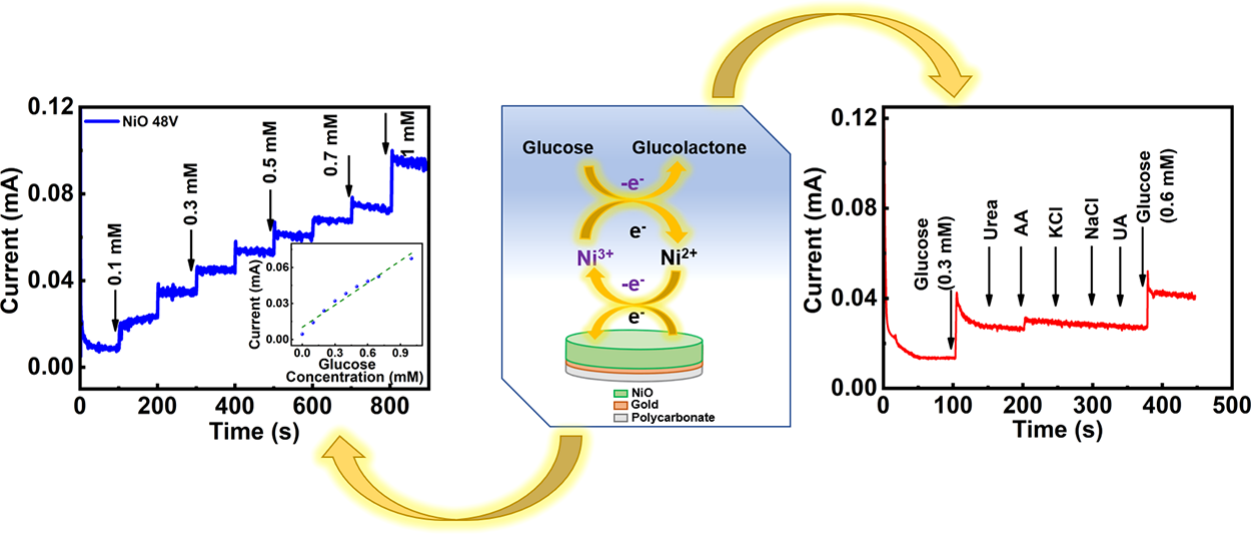

In this study, we successfully synthesized nickel oxide (NiO) nanoparticles (NPs), i.e., samples **NiO 24V**, **NiO 36V**, and **NiO 48V**, via an environmentally friendly one-step electro-exploding wire technique by employing three distinct voltage levels of 24, 36, and 48 V, respectively. Sample **NiO 48V** showed the most rugged surface and smallest particle size, which helped to enhance electrocatalytic properties. The highest Ni^3+^ content of sample **NiO 48V** contributed to the increasing redox current and rendering highly enhanced chemical reactions and thereby improving their electrochemical properties and electrocatalytic performance in the glucose oxidation processes in alkaline (0.1 M NaOH, pH = 13) media. The **NiO 48V** electrode showcased an excellent linear detection range spanning from 0.1 to 1 mM, featuring a remarkable sensitivity of 1202 μA mM^–1^ cm^–2^ and an exceptionally low limit of detection (LOD) value of 0.25 μM. Remarkably, NiO NPs exhibited exceptional long-term stability, commendable reproducibility, favorable repeatability, and outstanding selectivity. This study also highlights the excellent operational performance of the **NiO 48V** electrode in real-world samples, such as commercially available beverages and human urine, highlighting the practical nature of these nonenzymatic sensors in real-life scenarios for the food industries, clinical diagnostics, and biotechnology applications.

## 1 Introduction

Glucose, as an essential organic compound, is universally found in a diverse range of foods and beverages. It serves a purpose beyond basic sustenance, playing a crucial role in the human metabolism. This makes careful examinations of glucose levels essential for evaluating the consumable qualities and diagnosing specific metabolic disorders. Consequently, the assessments of the qualities of consumables and the diagnoses of specific metabolic disorders pivot heavily upon the precise analyses of glucose levels.^1,2^ Notably, high blood glucose levels constitute a hallmark characteristic of individuals afflicted with diabetes, a chronic medical condition troubled with a plethora of deleterious complications, including but not limited to cardiovascular diseases, renal dysfunctions, and ocular impairments.^3−5^ Given that foods and beverages signify the primary sources of glucose influx into the human body, the precise determination of glucose contents in these ingestible items assumes paramount importance in regulating the intake of this saccharide. Therefore, the need to ascertain both blood glucose levels and the glucose contents in foods and beverages arises as a critical imperative for the effective management of blood glucose levels.^6,7^ In pursuit of this objective, contemporary methodologies employed for the detection of glucose concentrations in biological fluids or edible items predominantly encompass colorimetry, fluorescence spectroscopy, and electrochemical sensors. Among these analytical techniques, electrochemical sensors stand out as the preeminent choice due to their widespread acceptance for their superior accuracies and effectiveness in glucose detection.^8,9^ Recent research has focused on developing fast, low-cost, noninvasive wearable electrochemical sensors for real-time glucose monitoring, combined with mobile health technology for efficient diabetes management. These wearable devices offer real-time, portable, and easy-to-use monitoring and are expected to replace traditional methods that are slower and require bulky equipment and trained personnel.^10^ Electrochemical sensors operate based on the principles of electrochemistry, employing electrodes to facilitate redox reactions with glucose molecules, thereby generating measurable electrical signals that are directly proportional to glucose concentrations. These glucose sensors offer advantages such as rapid response time, high sensitivities, and the potential for real-time monitoring, making them indispensable tools in clinical diagnostics and food quality assessments.^11−13^ Electrochemical glucose sensing can be categorized into enzymatic and nonenzymatic approaches. The enzymatic method relies on the immobilization of glucose oxidase on the electrode surface. However, this method faces challenges related to long-term stabilities due to the enzyme’s sensitivities to pH and temperature variations. Consequently, there are some significant interests reported in the literature for the development of nonenzymatic sensing techniques.^14,15^

Transition metal oxides such as Cu_2_O, CuO, NiO, Fe_2_O_3_, and Co_3_O_4_ have gained prominent attention in nonenzymatic glucose detection due to their robust electrochemical activities, chemical stabilities, and nontoxicities.^16−19^ These materials catalyze glucose electrochemical reactions, ensuring accurate quantification, and their biocompatibilities expand their utilities in analytical and biomedical applications, reinforcing glucose sensing research.^20−22^ In light of glucose detection, nickel oxide (NiO) is utilized because of its excellent electrochemical activities, stabilities, and safeties in comparison with other transition metal oxides, which catalyze glucose oxidations in electrochemical glucose sensors.^23−25^ Glucose undergoes oxidation due to the activity of NiO, releasing electrons. These electrons flow through the electrode to an external circuit, generating a current directly proportional to the glucose concentration.^26^ Nanoparticles have found broad application in electrochemical sensing platforms, owing to their exceptional catalytic activity, optical properties, and unique structural features.^27−29^ The catalytic activities, stabilities, affordabilities, high surface areas, and broad potential windows of NiO make it an ideal choice for sensitive and versatile glucose detections, particularly in wearable sensors.^12,30^ Among them, nickel (Ni) and nickel-based nanomaterials show remarkable electrocatalytic activities for glucose detection in alkaline environments, primarily due to the Ni(OH)_2_/NiOOH redox couple, wherein the redox reaction enables the direct oxidation of glucose to glucolactone. Furthermore, Ni-based nanomaterials are highly effective for nonenzymatic sensing, where the Ni(OH)_2_/NiOOH redox couple plays a crucial role.^31^ Several nonenzymatic glucose sensors have been developed by modifying substrates with various nickel (Ni) forms, including Ni nanoparticles (NPs) utilized in commercially available Ni forms for a glucose sensor with a linear range of 0.05–7.35 mM and a detection limit of 2.2 mM^32^ and loaded NiO on Ni forms for a sensor with a detection limit of 0.46 mM and a linear range of 0.005–5.5 mM.^33^

The electro-exploding wire (EEW) technique, while established, remains relatively unexplored in scientific circles. Its unique potential in various applications, notably in energy storage, catalysts, and sensors, warrants rigorous investigations. This technique’s precision in generating NiO NPs with tailored size and morphology holds promise for optimizing NiO’s electrochemical properties. Furthermore, the scalability and cost-effectiveness of the electro-exploding wire technique make it a promising avenue for the large-scale production of NiO NPs.^34,35^ In the realm of sensors, the sensitivities of NiO NPs generated via the electro-exploding wire technique make them ideal candidates for advanced sensor applications, enabling precise detection in diverse fields.^36^ Given these remarkable qualities, delving deeper into the details of the electro-exploding wire technique and further exploring its applications is essential. These explorations could lead to groundbreaking advancements across multiple domains, with NiO NPs serving as catalysts for transformative innovations.^37^

**Figure 1:**
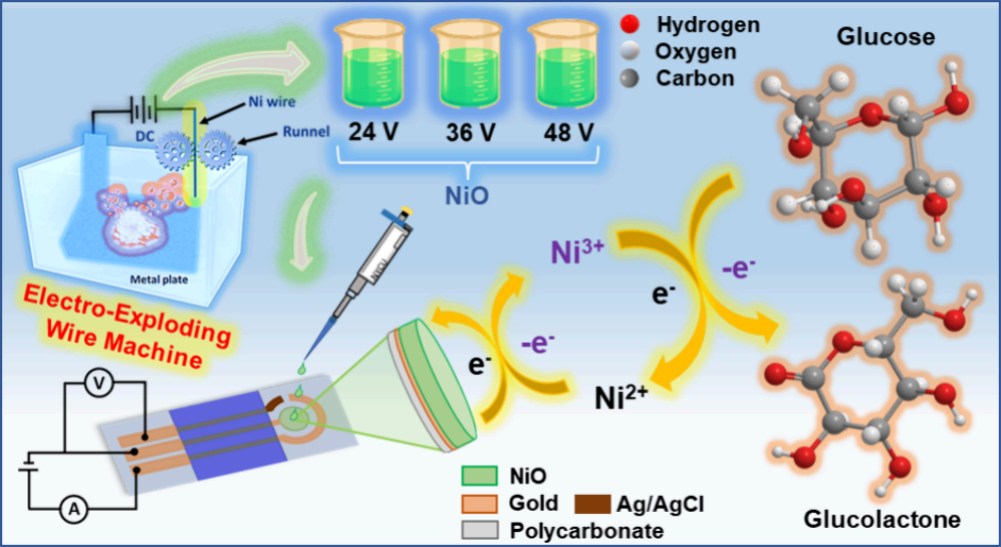
Schematic of synthesis steps of NiO NPs and the proposed reaction mechanism of **NiO 24V**, **NiO 36V**, and **NiO 48V** electrodes, corresponding to their exploding voltages of 24, 36, and 48 V, respectively.

In this work, we synthesized sensitive, simple transition metal oxide NiO NPs by the electro-exploding wire technique and deposited them on a flexible electrode as an electroactive transducer layer for the quantitative analysis of glucose in sodium hydroxide (NaOH) and beverages. The oxidation of Ni^2+^ to Ni^3+^ in an alkaline medium facilitates the oxidations of analytes, as shown in Figure 1, where glucose is oxidized to glucolactone. The **NiO 48V** electrode shows good electrocatalytic activity with excellent stability, linear response, and a low limit of detection (LOD) for glucose detection in NaOH medium. Moreover, the applied potential of 0.45 V allowed us to avoid the disturbance of the interference species that could contribute to the response of the **NiO 48V** electrode. **NiO 48V** electrode bending tests have shown high mechanical stabilities lasting 100 cycles of bending at 90°. Furthermore, the proposed electrochemical method has been successfully applied in the detection of glucose in real samples with excellent stabilities and reproducibilities.

## 2 Experimental Section

### 2.1 Materials

Nickel wires of 99% purity with a diameter of 0.3 mm were purchased from Nilaco Corporation, and d-(+)-glucose, ascorbic acid (AA), urea, uric acid (UA), and sodium chloride (NaCl) were purchased from Sigma-Aldrich. Potassium chloride (KCl) was purchased from Showa Chemical Company Limited. Sodium hydroxide (NaOH) was purchased from Choneye Pure Chemicals, Taiwan.

### 2.2 NiO Nanoparticle Synthesis

NiO nanoparticles (NPs) were synthesized in deionized water via the electro-exploding wire (EEW) technique by a nanoparticle generator (Metal Xano, Nanovie Co., Ltd.). The use of the EEW technique facilitates efficient and controlled synthesis, providing NPs with distinct characteristics.^38,39^ Four distinct direct-current (DC) voltages, specifically 24, 36, 48, and 72 V, were applied during these synthetic processes to create NP suspensions in 400 mL of deionized water. Nickel wires with a diameter of 0.3 mm and length of 74.6 cm were utilized in the NP generation process. The optimal conversion efficiency was about 30%, and then the concentration of the NiO suspension was carefully adjusted to be ∼0.006 M. The resulting NiO NPs were named as samples **NiO 24V**, **NiO 36V**, **NiO 48V**, and **NiO 72V**, corresponding to their exploding voltages of 24, 36, 48, and 72 V, respectively.

### 2.3 Materials Characterization

The structural and morphological characterizations of NiO NPs were examined via multiple analytical techniques. Transmission electron microscopy (TEM, JEOL ARM200F, Japan) was utilized to study the lattice structure and morphology of the NPs where the samples were prepared by drop-casting onto a copper mesh substrate. Samples prepared via spray coating on sapphire substrates were used for the analyses of scanning electron microscopy (SEM, FEI NOVA 200, USA) and atomic force microscopy (AFM, Dimension Edge, Bruker), which were employed to characterize the surface morphologies of the NPs. The crystalline structures of NiO NP powder samples were analyzed using X-ray diffraction (XRD, D8 Advance, Bruker AXS Inc., Germany) with Cu Kα radiation (λ = 1.54 Å) within a range of 2θ = 30°–70°. Furthermore, Raman scattering spectroscopy (MRI, ProTrustTech Co., Ltd.) was employed to characterize the NPs with a 532 nm laser excitation source, wherein NiO NP-stacked films were deposited on FTO/glass substrates using the spray coating machine.^38−40^ Fourier transform infrared spectra (FTIR) of spray-coated films were obtained using a PerkinElmer Spectrum 100 FTIR instrument. Samples of NiO NP-stacked films were prepared by using spray-coating on molded graphite laminates for the analyses of X-ray photoelectron spectroscopy (XPS, ESCALAB Xi+, Thermo Fisher Scientific, British), which provided information on the chemical compositions and valence states of the NPs.

### 2.4 Electrochemical Characterization

The electrochemical properties of **NiO 24V**, **NiO 36V**, and **NiO 48V** electrodes were investigated via a conventional three-electrode potentiostat system. Each electrode configuration employed a gold counter electrode, a Ag/AgCl reference electrode, and the NiO working electrode with a surface area of 0.0707 cm^2^. The flexible polycarbonate sensing electrode was produced by a conventional screen-printing method by ZensorSPE Taiwan, as shown in Figure S1a,b in the Supporting Information, prepared by drop casting 9 μL of **NiO** (**24**, **36**, and **48V**) suspension onto the substrate. A 0.1 M NaOH electrolyte served as the medium for all measurements. Cyclic voltammetry (CV) and amperometry (CA) measurements were conducted using an SP-50 potentiostat (BioLogic, France), and electrochemical impedance spectroscopy (EIS) was performed with an Autolab potentiostat (Metrhom, USA) within the same three-electrode setup.

## 3 Results and Discussion

### 3.1 Chemical Composition and Structural Characterization

**Figure 2:**
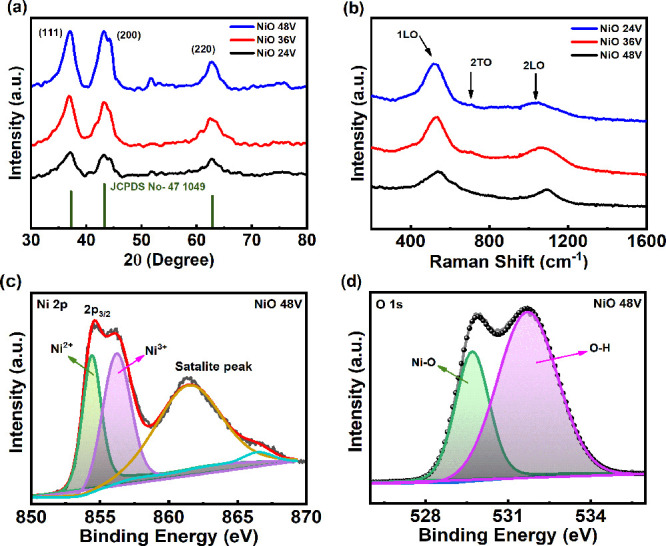
(a) X-ray diffraction (XRD) patterns of powder samples **NiO 24V**, **NiO 36V**, and **NiO 48V**. (b) Raman spectra of samples **NiO 24V**, **NiO 36V**, and **NiO 48V** by spray coating of NP-stacked films on FTO/glass substrates. X-ray photoelectron spectroscopy (XPS) spectra of (c) Ni 2p and (d) O 1s for sample **NiO 48V** were obtained by spray coating of NP-stacked film on the molded graphite laminate.

X-ray diffraction (XRD) spectra of samples **NiO 24V**, **NiO 36V**, and **NiO 48V** are shown in Figure 2a. The ideal XRD spectra of JCPDS No. 47-1049 for NiO are shown in Figure 2a as well, where the cubic structure of NiO belongs to the space group Fm3m with lattice constants of *a* = *b* = *c* = 4.19 Å. All samples of **NiO** (**24**, **36**, and **48V**) show identical crystalline structures similar to ideal NiO XRD spectra. This is strong evidence of the oxidation of nickel by electro-exploding in deionized water. Moreover, it can be observed that the peak intensity gradually increases with the increasing exploding voltage. In addition, we also observed that the position of the peak is shifted slightly toward the lower 2θ value. The position of the (111) peak shifted from an ideal 2θ of 37.25° to 37.14° in sample **NiO 24V**, whereas in samples **NiO 36V** and **NiO 48V**, the peak is shifted to 36.91° and 37.08°, respectively. This result indicates that the lattice spacing of the (111) plane is slightly increased to about 0.006, 0.021, and 0.010 Å for samples **NiO** (**24**, **36**, and **48V**). The crystallite size of all samples was calculated using Scherrer’s formula, eq 1.
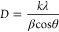
1The average crystallite sizes for samples of **NiO** (**24**, **36**, and **48V**) are estimated to be about 7.46, 7.02, and 6.58 Å, respectively. The details of peak position, lattice spacing, and full width at half-maximum (fwhm) are given in Table S1 in the Supporting Information.

The structural and chemical information on NiO NP-stacked films were studied using the Raman spectrum and shown in Figure 2b. The Raman scattering in NiO comes from one-phonon (LO and TO), two-phonon modes (2LO, 2TO, and LO+TO), magnon excitation (M), and some combination modes. The following Raman bands were observed for samples **NiO** (**24**, **36**, and **48V**). The stronger Raman peaks around 530.0 and 1050.0 cm^–1^ represent the first-order longitudinal (LO) mode and second-order longitudinal (2LO) of NiO that are related to the vibration of Ni–O bonds. The sharp emission around 530.0 cm^–1^ is attributed to the existence of Ni^3+^ defects.^41^ The second-order bands located at 712.7 cm^–1^ represent a transverse (2TO) mode.

X-ray photoelectron spectroscopy (XPS) was used to analyze the chemical composition of the samples **NiO** (**24**, **36**, and **48V**). All the data were calibrated to the C 1s peak position at 284.8 eV.^42^ It is observed from the survey spectra that in every sample, the Ni 2p_3/2_ peak along with the shakeup satellite peak is found within the range of 850 to 869 eV. In all the samples, there is no metallic Ni 2p_3/2_ peak, which should appear at 852.4 eV.^43^ This result confirms that the wire exploding technique is quite efficient in nickel oxidation. All of the Ni 2p_3/2_ peaks are fitted and separated into two peaks of Ni^2+^ and Ni^3+^ oxidation states. In the sample **NiO 48V**, the Ni^2+^ peak is located at 854.4 eV, whereas the Ni^3+^ peak is present at 856.2 eV, as illustrated in Figure 2c. XPS data of samples **NiO 24V** and **NiO 36V** are presented in Figure S2a,b in the Supporting Information. The Ni^2+^ and Ni^3+^ peaks of sample **NiO 24V** appear at 855.1 and 856.6 eV, whereas those of sample **NiO 36V** appear at 854.9 and 856.7 eV. The broad peaks observed at 861.6, 861.4, and 861.4 eV are related to the shakeup satellite of samples **NiO** (**24**, **36**, and **48V**), respectively. These shakeup satellite peaks of samples **NiO** (**24**, **36**, and **48V**) corresponding to their Ni^2+^ oxidation state were observed at 866.6 867.0, and 866.7 eV, respectively. These shakeup satellite peaks arose when electrons were excited to higher energy levels during their XPS photoemission processes.^44^

The existence of Ni^2+^ indicates the formation of NiO in the cubic rock salt structure.^45,46^ In addition, the presence of a significant amount of Ni^3+^ is observed, possibly due to the ionization of nickel vacancy and the presence of either Ni_2_O_3_ or NiOOH. However, earlier research reports that Ni_2_O_3_ is not stable in the ambient atmosphere and it absorbs the OH group from the atmosphere to form NiOOH. Most importantly, these NiO NPs are synthesized in deionized water, offering high possibilities of hydroxyl group absorption.^47^

Figure 2d gives XPS data of O 1s octahedron for sample **NiO 48V**. The peak at 529.7 eV is attributed to the lattice oxygen O bonded with Ni. Meanwhile, the peak located at 531.7 eV is denoted as OH bonded with Ni, as illustrated in Figure 2d. The same trend is revealed in XPS 1s data of samples **NiO 24V** and **NiO 36V,** as shown in Figure S2c,d in the Supporting Information. Nickel–oxygen bonding peaks in samples **NiO 24V** and **NiO 36V** are present at 529.90 and 530.02 eV, whereas the peaks at 531.63 and 571.70 eV are denoted as OH bonded with Ni. Further, to understand the predominant functional groups present in the samples **NiO 24V**, **NiO 36V**, and **NiO 48V**, Fourier transform infrared spectrum (FTIR) analysis was conducted at room temperature, as shown in Figure S3 in the Supporting Information. The FTIR spectra of all samples shown in Figure S3 demonstrated that the broad and intense band around 3400 cm^–1^ corresponds to the O–H stretching vibrations of interlayer water molecules and hydrogen-bonded OH groups.^48^ The peaks observed between 1000 and 1300 cm^–1^ are attributed to symmetric and asymmetric O–C=O stretching vibrations as well as C–O stretching, likely due to the adsorption of atmospheric CO_2_. Additionally, the lower absorption bands between 400 and 600 cm^–1^ are linked to metal–oxygen stretching vibrations, specifically associated with Ni–O.^49^ Overall, the FTIR spectra confirm the successful formation of NiO nanoparticles.

### 3.2 Characterization of Structure and Morphology

**Figure 3:**
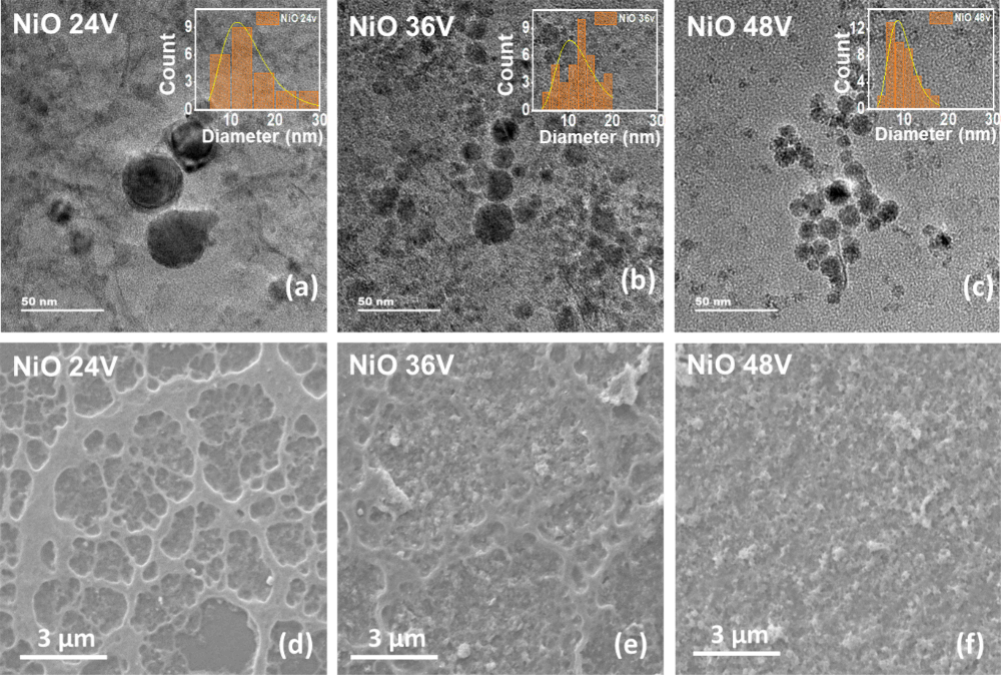
Transmission electron microscopy (TEM) images exhibiting morphologies of samples (a) **NiO 24V**, (b) **NiO 36V**, and (c) **NiO 48V** NPs by drop casting on copper mesh substrates, where the insets show particle size distributions. Scanning electron microscopy (SEM) images (top view) of samples (d) **NiO 24V**, (e) **NiO 36V**, and (f) **NiO 48V** by spray coating of NP-stacked films on sapphire substrates.

Transmission electron microscopy (TEM) images of NiO NPs synthesized at electro-exploding voltages of 24, 36, and 48 V are shown in Figure 3a–c. The insets exhibit the size distributions for all three synthesized suspensions at 24, 36, and 48 V with estimated average particle diameters of 12, 10, and 8 nm, respectively. The standard deviation of samples **NiO** (**24**, **36**, and **48V**) was 5.45, 3.98, and 3.02, and the wide size distribution ranges were 5.38–29.63, 4.12–20.01, and 4.12–17.98 nm, respectively, for NPs generated using different exploding voltages. The results of smaller NiO NPs at higher electro-exploding voltages are consistent with those in our previous research of WO_3_ NPs.^38^ Furthermore, the high-resolution TEM (HRTEM) images of NPs generated at various exploding voltages are presented in Figure S4a–c in the Supporting Information. Smaller crystallites are corroborated in HRTEM images that are in line with the crystallite size of ∼7 Å evaluated from the XRD spectra. The results indicate a polycrystalline structure in the NiO NPs.

The NiO NP suspensions are employed to deposit thin films on the sapphire substrate using a spray coating. The surface roughnesses of NiO films were analyzed using atomic force microscopy (AFM) in tapping mode with a scanning area of 5 × 5 μm^2^. It reveals root-mean-square surface roughnesses (*R*_q_) of 17.8, 34.9, and 64.8 nm for **NiO** (**24**, **36**, and **48V**) samples, respectively. The AFM pictorial representation in Figure S5a–c in the Supporting Information vividly depicts the surface roughness of the NiO samples, notably emphasizing that the sample **NiO 48V** film exhibits the largest surface roughness among all.

The scanning electron microscopy (SEM) images of samples **NiO** (**24**, **36**, and **48V**) were obtained for morphological analysis of NiO NP stacked films, as shown in Figure 3d–f. Sample **NiO 48V** exhibits a more dense, homogeneous, and continuous distribution of NiO NPs in comparison with those of other samples. In contrast, the sample **NiO 24V** film displays the lowest density with a noncontinuous feature. On the other hand, we present energy-dispersive X-ray spectroscopy (EDX) profiles of samples **NiO** (**24**, **36**, and **48V**) in Figure S6a–c in the Supporting Information. These SEM observations are consistent with our TEM and AFM analyses. The change of NP sizes and morphology variations of NP stacked films point to the important role played by the synthesis condition of the electro-exploding voltage. These nanomaterials can categorized by microscopy and other spectroscopic methods, and they have applications in optoelectronic, energy conversion, and storage fields.^50−52^

### 3.3 Electrochemical Characterization and Sensitivity Assessment

**Figure 4:**
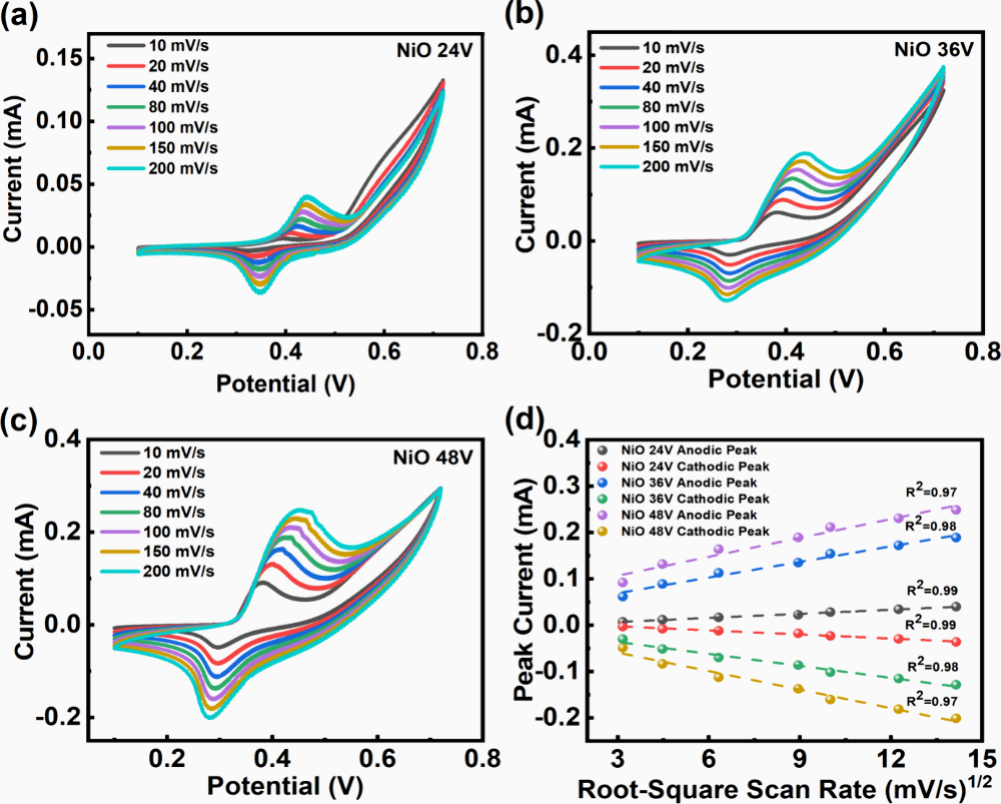
Cyclic voltammetry (CV) curves of (a) **NiO 24V**, (b) **NiO 36V**, and (c) **NiO 48V** electrodes in a mixed solution of 0.5 mM glucose and 0.1 M NaOH at scan rates ranging from 10 to 200 mV/s. (d) Calibration plots for both anodic and cathodic peak currents versus the square roots of the scan rates for all electrodes.

The electrochemical characteristics and electroanalytical mechanisms of all green synthesized NiO samples are studied by cyclic voltammetry (CV) before being pursued as a nonenzymatic sensor. Figure 4a–c shows the CV response for **NiO 24V**, **NiO 36V**, and **NiO 48V** electrodes in 0.5 mM glucose in 0.1 M NaOH. The comparison of cyclic voltammograms with and without 0.5 mM glucose (solid and dashed lines, respectively) is shown in Figure S7a–c in the Supporting Information. The changes in peak positions and peak currents in CV curves were observed in the presence of glucose. The shift in the peak position indicates that Ni^3+^ interacts with glucose, and the increased current suggests glucose oxidation. To understand the effects of pH values on the activities of electrodes, the CV measurements of NiO **48V** electrodes were carried out in acidic (0.1 M HCL, pH = 1), neutral (0.1 M PBS, pH = 7), and alkaline (0.1 M NaOH, pH = 13) media with and without 0.5 mM glucose (solid and dash lines, respectively) at the scan rate of 40 mV/s shown in Figure S8a–c in the Supporting Information, and significant redox current responses were only observed in the alkaline medium shown in Figure S8a. The alkaline medium enhanced the electrocatalytic activities of NiO, as shown in Figure S8a, which helped to oxidize NiO more easily and resulted in substantial changes in their redox peaks.^53^ Consequently, the alkaline media (0.1 M NaOH, pH 13) were utilized for our further investigations throughout this whole report. The electroactive surface areas for **NiO 24V**, **NiO 36V**, and **NiO 48V** in CV measurements were achieved as 0.0115, 0.0267, and 0.0871 cm^2^, respectively. The cyclic voltammetry response is recorded in the potential window from 0.1 to 0.7 V at various scan rates from 10 to 200 mV/s, as shown in Figure 4a–c. In all the electrodes, a pair of redox peaks can be observed, which implies good oxidation and reduction of glucose on NiO films. In the NP-stacked films, the nanoporous structure offers larger surface areas of NiO for water molecules and OH groups. It is argued that oxygen could drive a proton acceptor site for a water molecule through hydrogen bonding.^53^ Thus, it results in the generation of the hydroxyl phase on the surface of NiO such as the conversion from NiO to Ni(OH)_2_. The Ni(OH)_2_ oxidizes to NiOOH and, finally, glucose is oxidized to glucolactone by using NiOOH. These mechanisms are well discussed in previous papers.^15,53−55^

The reactions can be summarized as
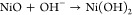

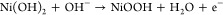

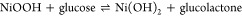


Herein, we can observe that glucose is catalytically oxidized by Ni^3+^ species to form glucolactone at the final step, pointing out the significant role of Ni^3+^ on the oxidization of glucose. The high valence state of Ni^3+^ plays a significant role in facilitating electron transfer by attracting the surrounding electrons. It promotes efficient charge transfer during the glucose oxidation process, enabling more effective electrochemical reactions, which is particularly beneficial in nonenzymatic glucose sensors, where the nickel species enhance the oxidation of glucose. The **NiO 48V** electrode consistently exhibits the highest redox peak current among all samples, regardless of scan rate or glucose concentration. Alternatively, the XPS analysis revealed various amounts of the Ni^2+^/Ni^3+^ couple across the samples. The enhanced peak current in sample **NiO 48V** may be attributed to the presence of different proportions of the Ni^2+^/Ni^3+^ redox couple in NiO. The area ratio of Ni^3+^ to Ni^2+^ was found to be 0.86, 0.96, and 1.86 for samples **NiO** (**24**, **36**, and **48V**), respectively, indicating the stoichiometric ratio of Ni^3+^ to Ni^2+^. The highest Ni^3+^ content was observed in sample **NiO 48V** in comparison to other samples, likely contributing to the increase in the peak redox current. This increase in Ni^3+^ content is associated with an increase in charge carriers and active sites for glucose oxidation, as glucose is oxidized by Ni^3+^ at the final step, as discussed earlier. Consequently, the presence of a large number of Ni^3+^ sites in the sample **NiO 48V** electrode could lead to the highest level of glucose oxidation.

To confirm the optimum detection of glucose oxidation by the **NiO 48V** electrode, the cyclic voltammetry (CV) response of the **NiO 72V** electrode is recorded as control experiments (for the higher exploding voltage synthesis of the **NiO** electrode) in the potential window from 0.1 to 0.7 V at various scan rates from 10 to 150 mV/s in 0.5 mM glucose in 0.1 M NaOH, as shown in Figure S9a. Regarding CV measurements, a pair of redox peaks can be observed that implies oxidation and reduction of glucose on NiO film. Figure S9b illustrates the amperometry (CA) curve of the **NiO 72V** electrode obtained in the sequential addition of glucose in a 0.1 M NaOH solution at a time interval of 100s. Notably, the current response did not show any sensitive response toward various concentrations of glucose (0.1–0.7 and 1 mM) at the applied potential of 0.45 V for glucose detection, due to the agglomeration of nanoparticles on the electrode which blocked the active surface site of the sensor. The X-ray diffraction (XRD) spectra for the **NiO 72V** sample are presented in Figure S9c. For reference, the ideal XRD pattern of NiO, based on JCPDS No. 47-1049, is displayed in Figure 2a. This pattern corresponds to a cubic structure with a space group of *Fm*3*m* and lattice constants of *a* = *b* = *c* = 4.19 Å. The **NiO 72V** sample exhibits a crystalline structure nearly identical to the ideal NiO pattern, providing strong evidence of nickel oxidation through electro-explosion in deionized water. The SEM images (top view) of **NiO 72V** shown in Figure S9d indicate that the **NiO 72V** formed surface aggregates due to agglomeration of NiO nanoparticles, which impaired its sensitivity toward glucose detections. Thus, it has been verified that the **NiO 48V** electrode possessed the best sensitivity toward glucose.

In previous paragraphs, we presented morphological analyses using SEM and AFM and observed that sample **NiO 48V** exhibits the densest and most homogeneous film with the roughest surface in comparison to other samples. It is established that rough surfaces, as compared to smoother counterparts, generally offer larger surface areas. The expanded surface areas pave the way for increased active and adsorption sites, thereby establishing a conducive interface for efficient electron transfer during electrochemical reactions. In this context, a rougher surface may enhance the sensitivity of the sensors by facilitating intensive interaction between the sensor surface and the target analyte.^56^ The morphology of sample **NiO 48V** corroborates a higher density of active sites for glucose oxidation in the **NiO 48V** electrode, further supporting the observation of the highest redox peak current in this electrode. In a similar way, we can conclude the enhanced peak current in the **NiO 36V** electrode rather than in the **NiO 24V** electrode.

It has been observed from the different scan rate measurements that the separation potential between the two redox peaks increases with an increasing scan rate. Both of the redox peaks are shifted toward higher potential with an increasing scan rate. Also, the peak intensity increases with an increasing scan rate. The peak current vs the square root of the scan rate curve in Figure 4d shows a linear relation, indicating that the electrode reaction is diffusion-controlled. It can be inferred from all of the analyses that the electrode kinetics is quasi-reversible in nature. To inspect the stability of the electrode, five consecutive cycles are measured by CV at a fixed scan rate (20 mV/s) with the other conditions fixed shown in Figure S10a–c in the Supporting Information. All the voltammograms are well overlapped, implying superior stability of all NiO electrodes.^57^

### 3.4 Glucose Sensing Performance According to Amperometry (CA)

**Figure 5:**
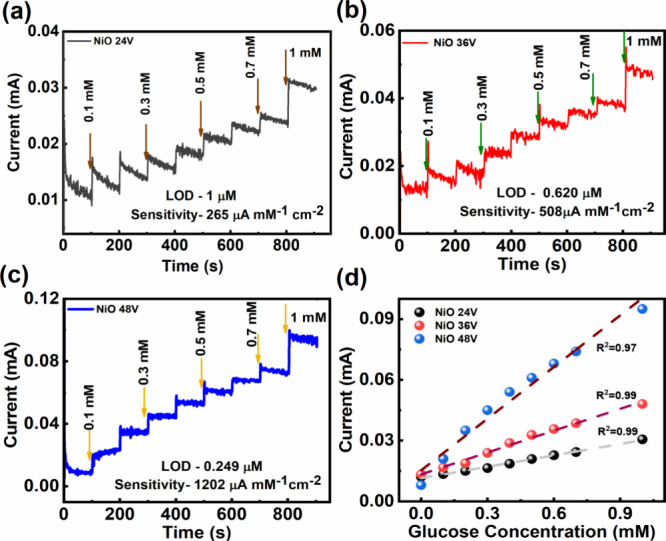
Glucose sensing performance of amperometry (CA) measurements for (a) **NiO 24V**, (b) **NiO 36V**, and (c) **NiO 48V** electrodes at a potential of 0.45 V, where the reference electrode is Ag/AgCl in mixed solutions of 0.1 M NaOH containing various glucose concentrations ranging from 0 to 1 mM. (d) Calibration curves are calculated from Figure 5a–c for three electrodes, where the dashed lines give the best linear least-squares fittings for the correlations between the current and the glucose concentration.

Following a comprehensive analysis of the electrochemical behavior of **NiO 24V**, **NiO 36V**, and **NiO 48V** electrodes, all the necessary parameters are determined for the subsequent chronoamperometric detection of glucose. Figure 5a–c illustrates amperometry curves obtained in the sequential addition of glucose in a 0.1 M NaOH solution for **NiO** (**24**, **36**, and **48V**) electrodes, respectively. Notably, the current response exhibits the familiar stairs-like behavior regarding the concentration of glucose. The applied potential for glucose detection is set at 0.45 V for all of the fabricated electrodes. Figure 5d also presents the linear calibration curves derived from those stairs-like amperometry curves, demonstrating the sensitive responses between currents and glucose concentrations. The sensitivities of **NiO** (**24**, **36**, and **48V**) electrodes toward glucose are determined from the slopes of the calibration curves and are estimated to be 265, 508, and 1202 μA mM^–1^ cm^–2^, respectively. The current responses for **NiO** (**24**, **36**, and **48V**) electrodes showed a good linear relationship within the added glucose concentrations ranging from 0.1 to 0.7 mM (with a span of 0.1 mM), and then to 1 mM (with a span of 0.3 mM), described by *y* = 0.0187*x* + 0.0114 (*R*^2^ = 0.99), *y* = 0.0359*x* + 0.0134 (*R*^2^ = 0.99), and *y* = 0.0851*x* + 0.0153 (*R*^2^ = 0.97). The response time of the sensor is 3 s for both lower and higher concentrations of glucose, as shown in Figure S11a,b in the Supporting Information. The limit of detection (LOD) values are calculated using eq 2.^58,59^
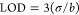
2

In the equation, σ signifies the standard deviation of the blank measurements, and *b* denotes the slope of the calibration curve. The calculated LOD values are found to be 1.00, 0.62, and 0.25 μM for **NiO** (**24**, **36**, and **48V**) electrodes, respectively. Electrode sensitivities, limit of detection (LOD) values, and linear ranges of enzyme-based glucose sensors published in related references are compared in Table S3 in the Supporting Information, which give moderate sensitivity and LOD values in contrast to those nonenzymatic glucose sensors shown in Table S2 with larger variations of sensitivity and LOD values. Both types of enzyme-based glucose sensors and nonenzymatic glucose sensors frequently reveal various linear ranges for glucose detection. Accordingly, our proposed sensor **NiO 48V** displayed good sensitivity, detection of limit, and linear range toward glucose detection among nonenzymatic glucose sensors shown in Table S2 and enzyme-based glucose sensors shown in Table S3, which can also be utilized multiple times in comparison with enzyme-based glucose sensors with limitations to temperatures and humidity levels.

### 3.5 Selectivity and Long-Term Stability Test

**Figure 6:**
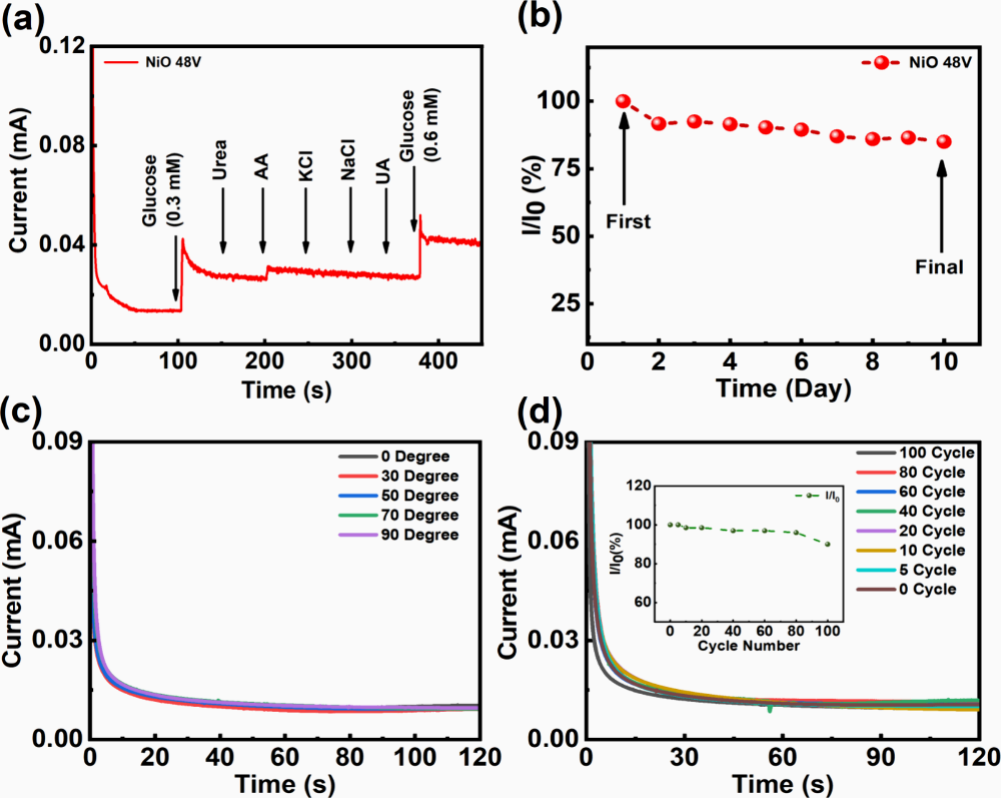
Sensing performance of the **NiO 48V** electrode for (a) electroanalytical selectivity tests for glucose detections with multiple interfering agents, (b) long-term stability and reusability tests up to 10 days, (c) flexibility tests after multiple bending angles (0°, 30°, 50°, 70°, and 90°), and (d) stability tests after 100 bending cycles at the bending angle of 90°.

Selectivity is the most important and primary characteristic of sensors, and it shall be examined under the conditions of various interfering agents in the analyte of interest. Figure 6a illustrates selectivity tests conducted on the **NiO 48V** electrode, aiming to detect 0.3 and 0.6 mM of glucose amidst the interference of several different agents, including 0.1 mM ascorbic acid (AA), 0.5 mM urea, 50 μM uric acid (UA), 10 mM NaCl, and 10 mM KCl. The **NiO 48V** electrode exhibits extremely high selectivity and detection of glucose possibly due to a specific interaction between glucose and the **NiO 48V** sample. Figure 6b shows the long-term stability of the **NiO 48V** electrode for glucose detection over the course of 10 days of the experimentation. Following each daily experiment, the **NiO 48V** electrode is refreshed and rinsed in 0.1 M NaOH solution with deionized water. The **NiO 48V** electrode is then kept at room temperature for subsequent tests. The maximum deviation in current is approximately 15% for up to 10 days, which has a minimal impact on the sensitivity of the **NiO 48V** electrode. After 10 days of consecutive measurements, we analyzed the **NiO 48V** sample by using X-ray photoelectron spectroscopy (XPS), X-ray diffraction (XRD), and scanning electron microscopy (SEM). As shown in Figure S12a,b in the Supporting Information, XPS spectra for Ni 2p and O 1s reveal that the Ni^2+^ peak remained stable at 855.2 eV, while the Ni^3+^ peak endured at 856.2 eV, as mentioned in Figure 2c and prior analyses. However, the Ni^3+^/Ni^2+^ area ratio decreased slightly from 1.86 to 1.20, suggesting a modest reduction in the Ni^3+^ content due to repeated electrode usage. The XRD analyses showed no significant changes in the diffraction pattern of **NiO 48V** after 10 days, as shown in Figure S12c, indicating that the sample maintained its structural integrity. The peaks were sharp and consistent when compared to the initial measurement in Figure 2a. The SEM images from Figures 3f and S12d (before/after measurements, respectively) showed a slight change of surface morphology with lower porosities due to the repeated measurements and glucose depositions on the surface, confirming the strong structural stability of the **NiO 48V** sample over a period of 10 days. This indicates that **NiO 48V** retains its structural and chemical stabilities, even after extended usage.

### 3.6 Sensor Performance under Different Angles of Bending

To evaluate their viability for wearable electronics, the mechanical flexibility of the **NiO 48V** electrode is examined. As depicted in Figure S13a–d in the Supporting Information, the **NiO 48V** electrode exhibits remarkable flexibility, capable of bending at angles from 0° to 90° without any significant degradation in its response of current (see Figure 6c). Even after 100 cycles of bending at 90°, the **NiO 48V** electrode retains ∼90% of its original response current, as illustrated in Figure 6d. This outstanding mechanical flexibility and stability empower the newly developed **NiO 48V** electrode for potential applications in flexible and wearable technologies.

### 3.7 Electrochemical Impedance Spectroscopy Test

Electrochemical impedance spectroscopy (EIS) was employed to assess the efficacies of all electrodes. The EIS measurement is performed in an alternating current (AC) at frequencies ranging from 0.1 to 100 kHz. The oscillation amplitude is fixed at the *V*_rms_ of 10 mV and a working potential of 0.5 V with regard to the Ag/AgCl reference electrode. The Nyquist plot in Figure S14a in the Supporting Information illustrates the impedance characteristics of **NiO 24V**, **NiO 36V**, and **NiO 48V** electrodes immersed in the 0.1 M NaOH aqueous solution. The Nyquist plots exhibit a distinctive pattern comprising a depressed semicircle succeeded by an inclined straight line. This depressed semicircle is indicative of the double-layer capacitance and charge transfer resistance.^60^ In the low-frequency range, the straight line corresponds to ion diffusion, which is known as the Warburg element.^60^Figure S14b in the Supporting Information illustrates the equivalent circuit aligned with the Nyquist plot, facilitating the derivation of accurate impedance values. To account for the depressed semicircle observed, it becomes essential to substitute the capacitor in the equivalent circuit with a constant phase element (CPE). Within this electrical equivalent circuit, *R*_s_, *Q*_dl_, *R*_ct_, and *W* denote the solution resistance, double-layer capacitance, charge transfer resistance, and Warburg element, respectively. Specifically, the charge transfer resistance associated with the faradic reaction on the electrode serves as the circuit element conveying a straightforward physical interpretation of the transfer of charge at the electrode–electrolyte interface.^60^ The corresponding values for *R*_ct_ are 279.8, 10.29, and 5.1 Ω for **NiO** (**24**, **36**, and **48V**) electrodes, respectively. The **NiO 48V** electrode, characterized by the lowest charge transfer resistance, signifies the swiftest rate of charge transfer in the electrochemical reaction, indicating a faster faradic reaction compared to the other two samples. This observation is in line with the cyclic voltammetry data, where the **NiO 48V** electrode exhibits the highest anodic current peak. Conversely, the **NiO 24V** electrode displays the lowest anodic current peak across all scan rates, accompanied by a substantial charge transfer resistance value. In the low-frequency domain, the straight line signifies the presence of the Warburg element, reflecting the diffusion process. Notably, **NiO 36V** and **NiO 48V** electrodes exhibit a more pronounced and steeper slope, while the **NiO 24V** electrode displays a comparatively gentler slope. The steeper slope implies a swifter diffusion of ions participating in the electrochemical process, whereas a shallower slope indicates a slower diffusion process in the case of the **NiO 24V** electrode.^61^

### 3.8 Glucose Detection of Beverage Products and Human Urine via Amperometry (CA) Measurements

**Figure 7:**
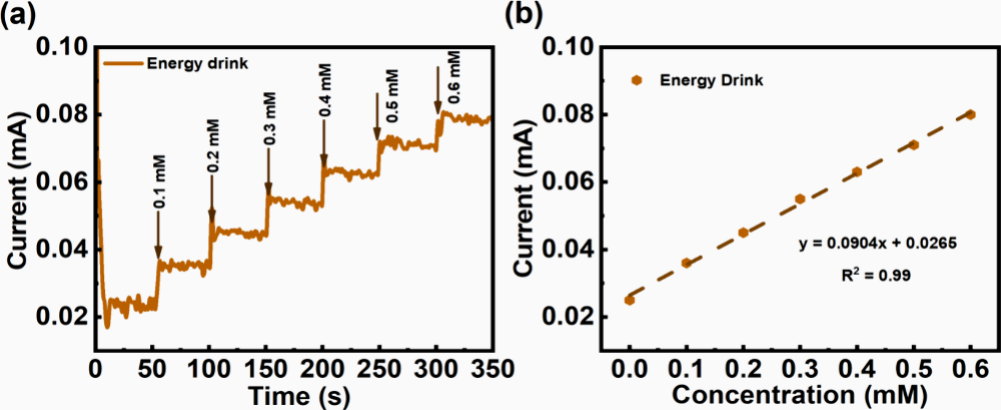
(a) Amperometry (CA) measurements of the **NiO 48V** electrode with sequential additions of 0.1 mM glucose in 0.1 M NaOH solutions containing energy drink at 0.45 V. (b) Calibration curve for the fabricated electrode, where the dashed line gives the best linear least-squares fitting for the correlation between the current and the glucose concentration.

To evaluate the potential applications of the fabricated electrode, the **NiO 48V** electrode was implemented to detect glucose in market available beverages, such as energy drink, guava tea, fruit juice, and human urine, which were prediluted 100 times in 0.1 M NaOH solution and subsequently tested using amperometry (CA) at 0.45 V. Remarkably, the detection results of glucose CA measurements of energy drink are presented in Figure 7a; the current responses for energy drink showed a good linear relationship within the added glucose concentrations ranging from 0.1 to 0.6 mM, described by *y* = 0.0904*x* + 0.0265 (*R*^2^ = 0.99) in Figure 7b. Similarly, the glucose detection measurements of guava tea, fruit juice, and human urine are presented in Figure S15a,c,e in the Supporting Information, and the current response for guava tea, fruit juice, and human urine is described by *y* = 0.0612*x* + 0.0356 (*R*^2^ = 0.96), *y* = 0.0917*x* + 0.0503 (*R*^2^ = 0.98) and *y* = 0.0949*x* + 0.0237 (*R*^2^ = 0.99) in Figure S15b,d,f in the Supporting Information, respectively. This demonstrates that the **NiO 48V** electrode effectively senses glucose levels in these real beverage samples and human urine. The recoveries were calculated for energy drinks, guava tea, fruit juice, and human urine by the CA responses, as shown in Table 1. The recovery of the beverage products ranged from 92.00 to 107.43%, with a relative standard deviation (RSD) of less than 6%. These results corroborate that the developed sensors can reliably detect glucose contents in real beverage samples and human urine, thereby presenting a remarkable strategy for monitoring glucose in food products and human urine.^14^

**Table 1:** Analytical Results of Glucose Detection for the Spiked Samples of Available Beverages (Including Energy Drink, Guava Tea, Fruit Juice, and Human Urine) on the **NiO 48V** Electrode^a^

sample^b^	glucose spiked (mM)	glucose found (mM)	RSD (%)	recovery (%)
energy drink	0.1	0.105	4.21	105.00
	0.3	0.320	2.78	106.67
	0.5	0.490	2.11	98.00
guava tea	0.1	0.104	3.69	104.57
	0.3	0.316	2.75	105.66
	0.5	0.464	2.35	92.81
fruit juice	0.1	0.094	3.39	94.47
	0.3	0.321	2.44	107.28
	0.5	0.476	2.14	95.28
deionized water	0.1	0.102	5.71	102.06
	0.3	0.301	3.28	100.65
	0.5	0.537	2.45	107.43
human urine	0.1	0.092	3.12	92.00
	0.3	0.290	2.31	96.67
	0.5	0.507	2.18	101.4

a Beverage samples were prediluted 100 times in 0.1 M NaOH solutions and subsequently tested with the **NiO 48V** electrode using amperometry (CA) at 0.45 V. RSD(%): relative standard deviation for three consecutive measurements.

b All samples are prediluted 100 times in 0.1 M NaOH solutions. Glucose contents in the energy drink, guava tea, and fruit juice were 0.07 g/mL, 0.11 g/mL, and 0.10 g/mL, respectively.

## 4 Conclusions

In summary, we successfully synthesized NiO nanoparticles (NPs), i.e., samples **NiO 24V**, **NiO 36V**, and **NiO 48V**, via the electro-exploding wire technique by employing three distinct voltage levels of 24, 36, and 48 V, respectively. Since NiO films have been widely utilized for glucose detections by cyclic voltammetry (CV) and amperometry (CA) measurements, good oxidation and reduction peaks in CV responses at various scan rates (10 to 200 mV/s) could be characterized on **NiO 24V**, **NiO 36V**, and **NiO 48V** electrodes, which were prepared by NiO NPs (i.e., samples **NiO 24V**, **NiO 36V**, and **NiO 48V**) formed under corresponding potentials of 24, 36, and 48 V. Notably, the **NiO 48V** electrode demonstrated the most superior electrochemical catalytic activity in glucose oxidation in the CV measurements in alkaline (0.1 M NaOH, pH = 13) media with the substantial presence of a redox couple Ni^2+^/Ni^3+^, which could be attributed to the highest Ni^3+^ content of sample **NiO 48V** observed in X-ray photoelectron spectroscopy (XPS) measurement resulting in the highest peak currents in the CV response of **NiO 48V** electrode with an increase in charge carriers and active sites for glucose oxidation. Moreover, **NiO 48V** showed the most rugged surface (*R*_q_ = 64.8 nm), the smallest particle size (8 nm), and dense active site/maximum area coverage among all samples in atomic force microscopy (AFM), transmission electron microscopy (TEM), and scanning electron microscopy (SEM) images, respectively. The current responses of CA curves of all electrodes obtained by sequential additions of glucose in NaOH solution exhibit similar stair-like behaviors regarding the concentrations of glucose at an applied potential of 0.45 V for all fabricated electrodes. Accordingly, the **NiO 48V** electrode displayed the optimum sensitivity of 1202 μA mM^–1^ cm^–2^, with a remarkably lowest limit of detection (LOD) value of 0.25 μM. Furthermore, the **NiO 48V** electrode showcased an outstanding selectivity for glucose, as the presence of various interfering agents (including real beverage samples) did not have obvious impacts on the device's performance. The longevity study over a 10-days revealed a consistent and stable sensor response. Additionally, a mechanical stability test involving 100 bendings at a 90° angle demonstrated no significant performance loss, featuring the device's robust performance as an excellent flexible nonenzymatic glucose sensor. These findings indicate that our NiO NPs synthesized via the electro-exploding wire technique exhibit superior operational performance, particularly in real-world samples like commercially available beverages and human urine, which underscores the practicality of this nonenzymatic sensor in various fields, such as the food industry and clinical diagnostic applications.

## Supporting Information Available

Additional characterization data (CV, CA, EIS, XPS, FTIR, AFM, TEM, SEM, EDX, and XRD) and comparison table (PDF)
