# Supplementary material for: Flexible Non-Enzymatic Glucose Sensors: One-Step Green Synthesis of NiO Nanoporous Films via an Electro-Exploding Wire Technique
Source: ACS Appl Mater Interfaces. 2024 Nov 12;16(47):64494–504. doi: 10.1021/acsami.4c13653 (PMC11615849; doi:10.1021/acsami.4c13653)
Supplement: Supplementary file 1 — am4c13653_si_001.pdf [file am4c13653_si_001.pdf]

Supporting Information

Flexible Non-Enzymatic Glucose Sensors: One-Step Green Synthesis of NiO Nanoporous Films via Electro-Exploding Wire Technique

Nadeem Ahamad,[a] Soumallya Banerjee,[a] Chia-Chun Wei,[b] Kuan-Cheng Lu,[b] Akhil Pradiprao Khedulkar,[b] Wen-Bin Jian,[b] Sadiq Mahmood,[c] Chih-Wei Chu,[d] Hong-Cheu Lin[a,e]**

[a] Department of Materials Science and Engineering, National Yang Ming Chiao Tung University, Hsinchu 300093, Taiwan.

[b] Department of Electrophysics, National Yang-Ming Chiao Tung University, Hsinchu 300093, Taiwan.

[c] International College of Semiconductor Technology, National Yang Ming Chiao Tung University, Hsinchu 300093, Taiwan.

[d] Research Center for Applied Sciences, Academia Sinica, Taipei, 11529, Taiwan.

[e] Center for Emergent Functional Matter Science, National Yang Ming Chiao Tung University, Hsinchu 300093, Taiwan.

Corresponding Authors

Wen-Bin Jian - Department of Electrophysics, National Yang-Ming Chiao Tung University, Hsinchu 300, Taiwan; Email: wbjian@nycu.edu.tw

Hong-Cheu Lin - Department of Materials Science and Engineering; Center for Emergent Functional Matter Science, National Yang Ming Chiao Tung University, Hsinchu 300093, Taiwan; Email: linhc@nycu.edu.tw

Table S1. A list of Miller Indices, 2θ peak positions, d-spacing values, and full width half maxima (FWHM) of XRD data measured by powder samples of **NiO 24V**, **NiO 36V**, and **NiO 48V**.

Sample	Miller Index	2ϴ (Degree)	d Spacing (Å)	FWHM (Degree)
**NiO 24V**	111	37.14	2.42	0.023
	200	43.21	2.10	0.025
	220	62.75	1.48	0.022
**NiO 36V**	111	36.91	2.43	0.021
	200	43.23	2.09	0.024
	220	62.43	1.48	0.019
**NiO 48V**	111	37.08	2.42	0.022
	200	43.21	2.09	0.029
	220	62.75	1.48	0.023

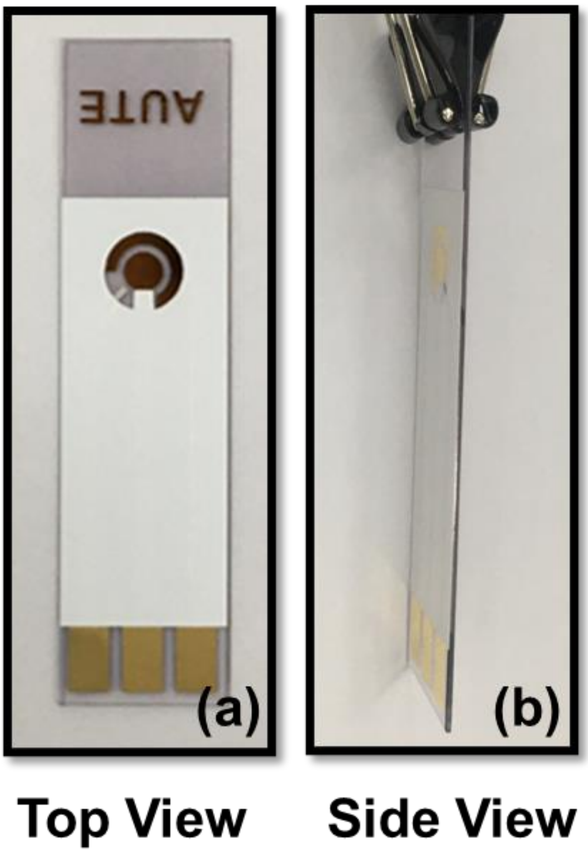

Figure S1. Photo images of the conventional screen-printed gold electrode taken by **(a)** top view and **(b)** side view.

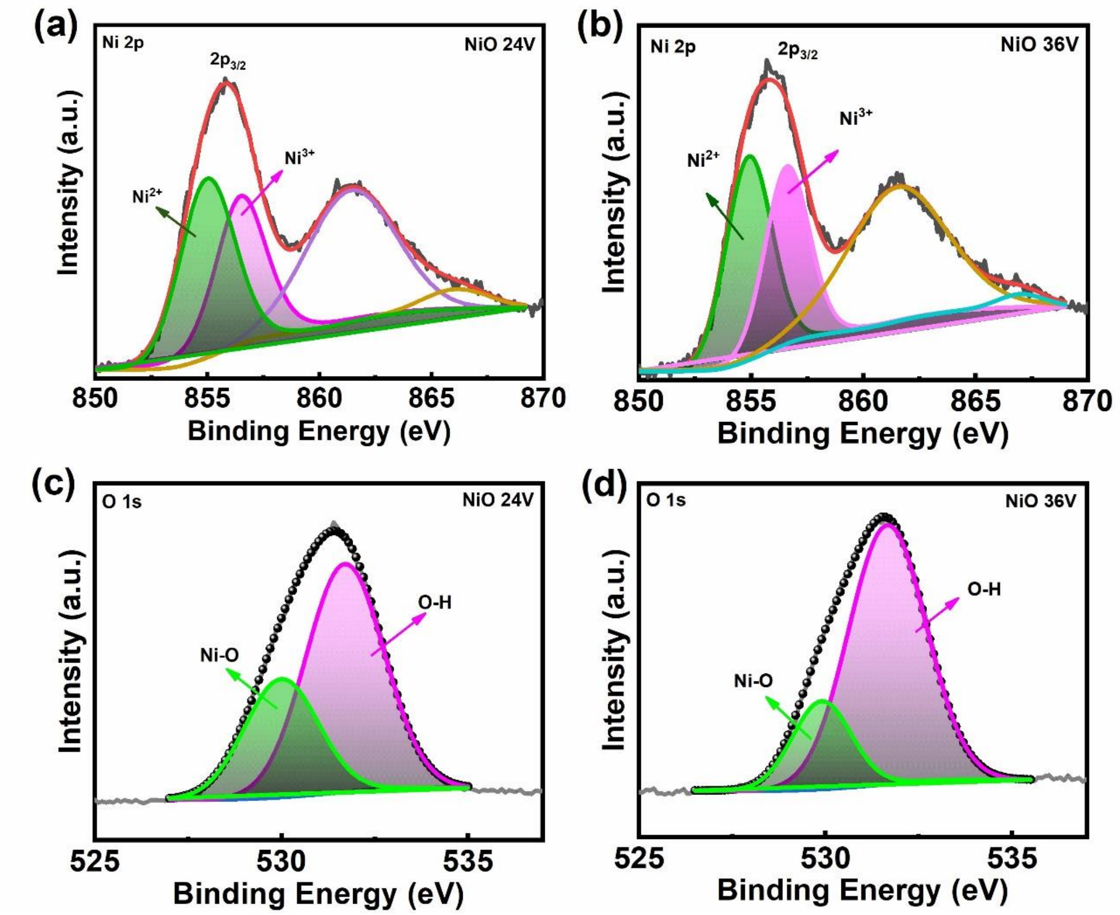

Figure S2. X-ray photoelectron spectroscopic (XPS) data of Ni 2p spectra for samples **(a) NiO 24V** and **(b) NiO 36V**, along with O 1s spectra for samples **(c) NiO 24V** and **(d) NiO 36V** by spray-coating on molded graphite laminates.

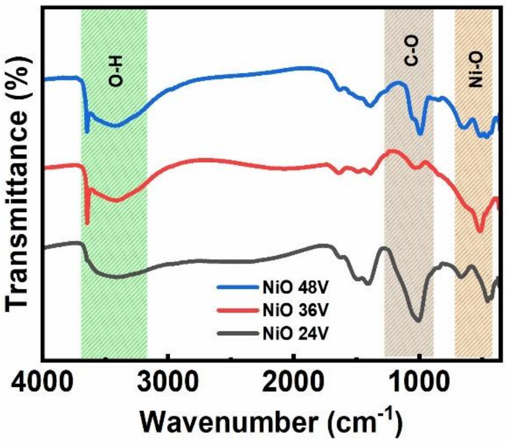

Figure S3. Fourier transform infrared spectrum (FTIR) of samples **NiO 24V**, **NiO 36V**, and **NiO 48V** by spray coating onto a silicon substrate.

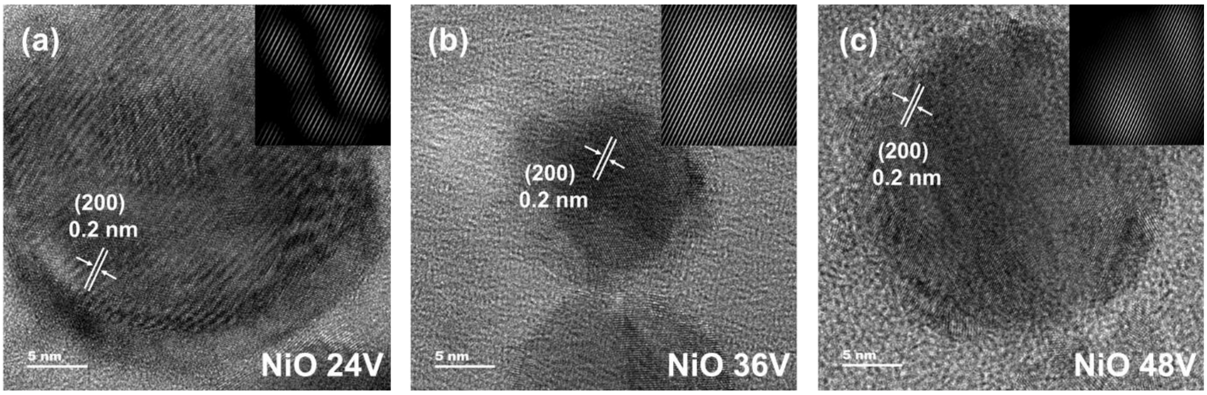

Figure S4. High-resolution transmission electron microscope (HRTEM) images of samples **(a) NiO 24V**, **(b) NiO 36V**, and **(c) NiO 48V** by drop-casting onto a copper mesh substrate.

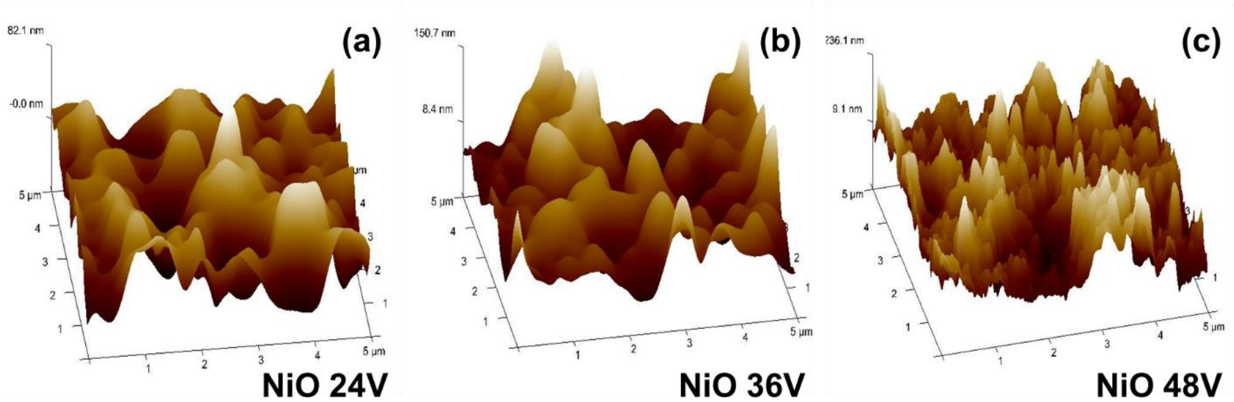

Figure S5. Atomic force microscope (AFM) images of samples **(a) 24V NiO**, **(b) 36V NiO**, and **(c) 48V NiO** via spray coating on sapphire substrates.

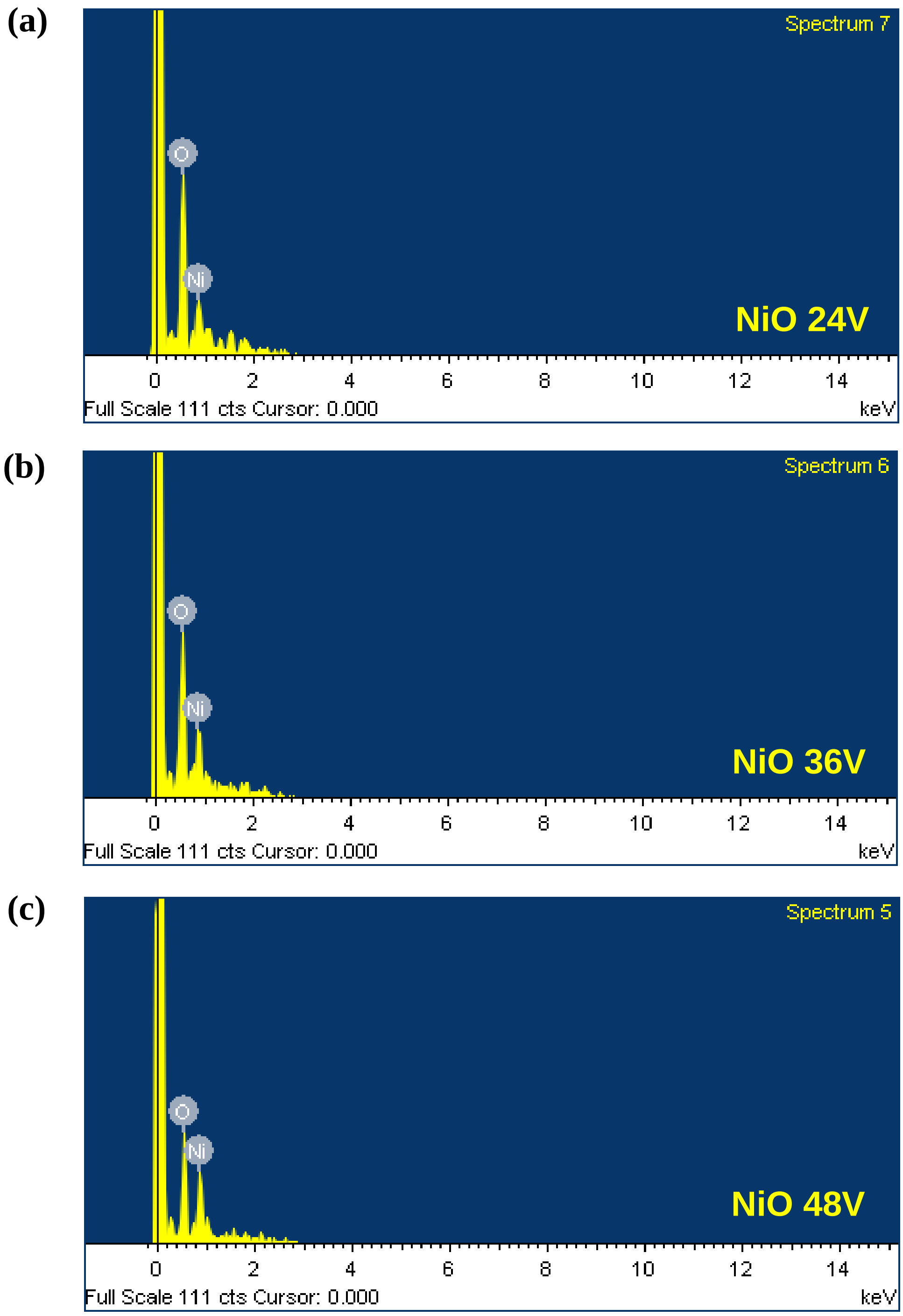

Figure S6. Energy-dispersive X-ray spectroscopy (EDX) profiles of samples **(a) NiO 24V**, **(b) NiO 36V**, and **(c) NiO 48V** via spray coating on sapphire substrates.

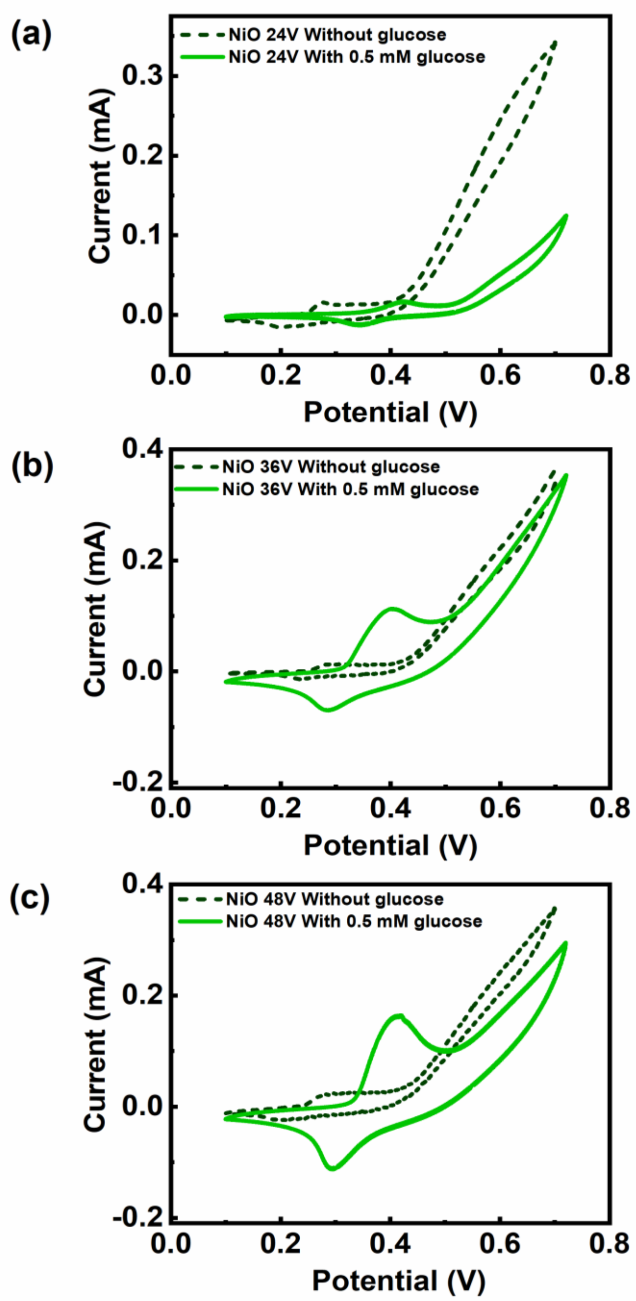

Figure S7. Cyclic voltammetry (CV) curves of **(a) NiO 24V**, **(b) NiO 36V**, and **(c) NiO 48V** electrodes with and without 0.5 mM glucose (solid and dash lines, respectively) at a scan rate of 40 mV/s.

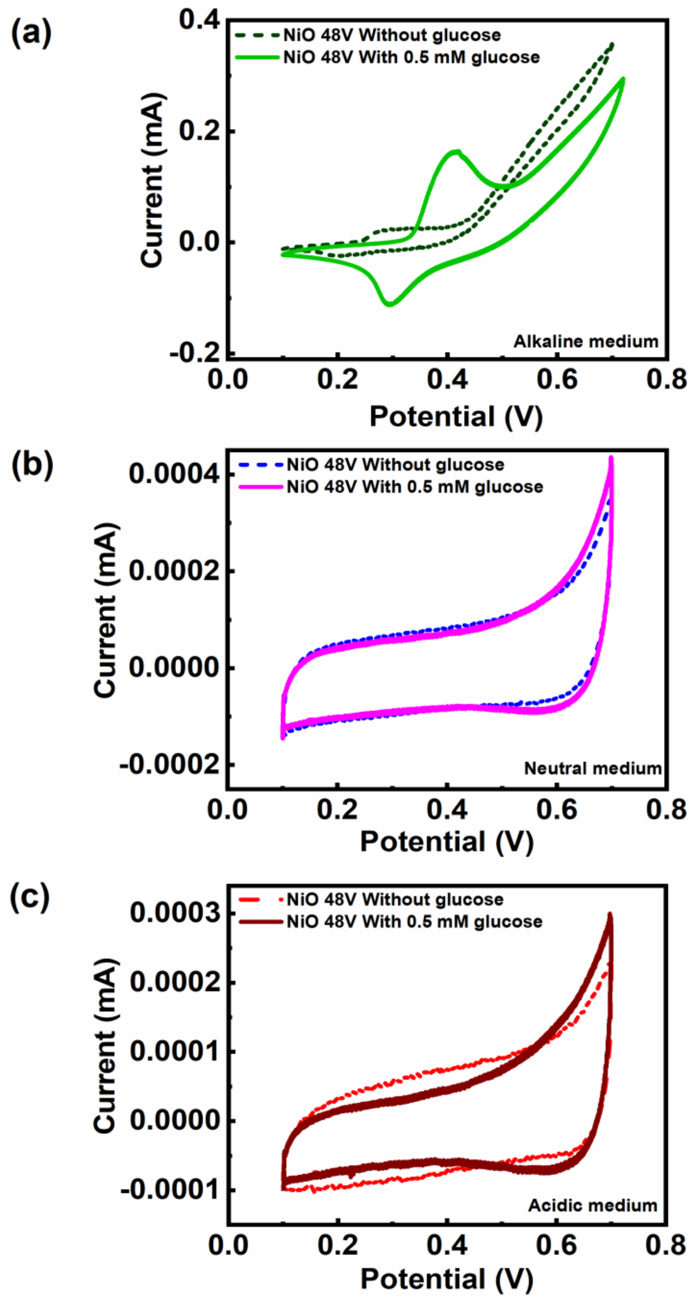

Figure S8. Cyclic voltammetry (CV) curves of **NiO 48V** electrodes in **(a)** alkaline (0.1 M NaOH, pH=13), **(b)** neutral (0.1 M PBS, pH=7), and **(c)** acidic (0.1 M HCL, pH=1) media with and without 0.5 mM glucose (solid and dash lines, respectively) at a scan rate of 40 mV/s.

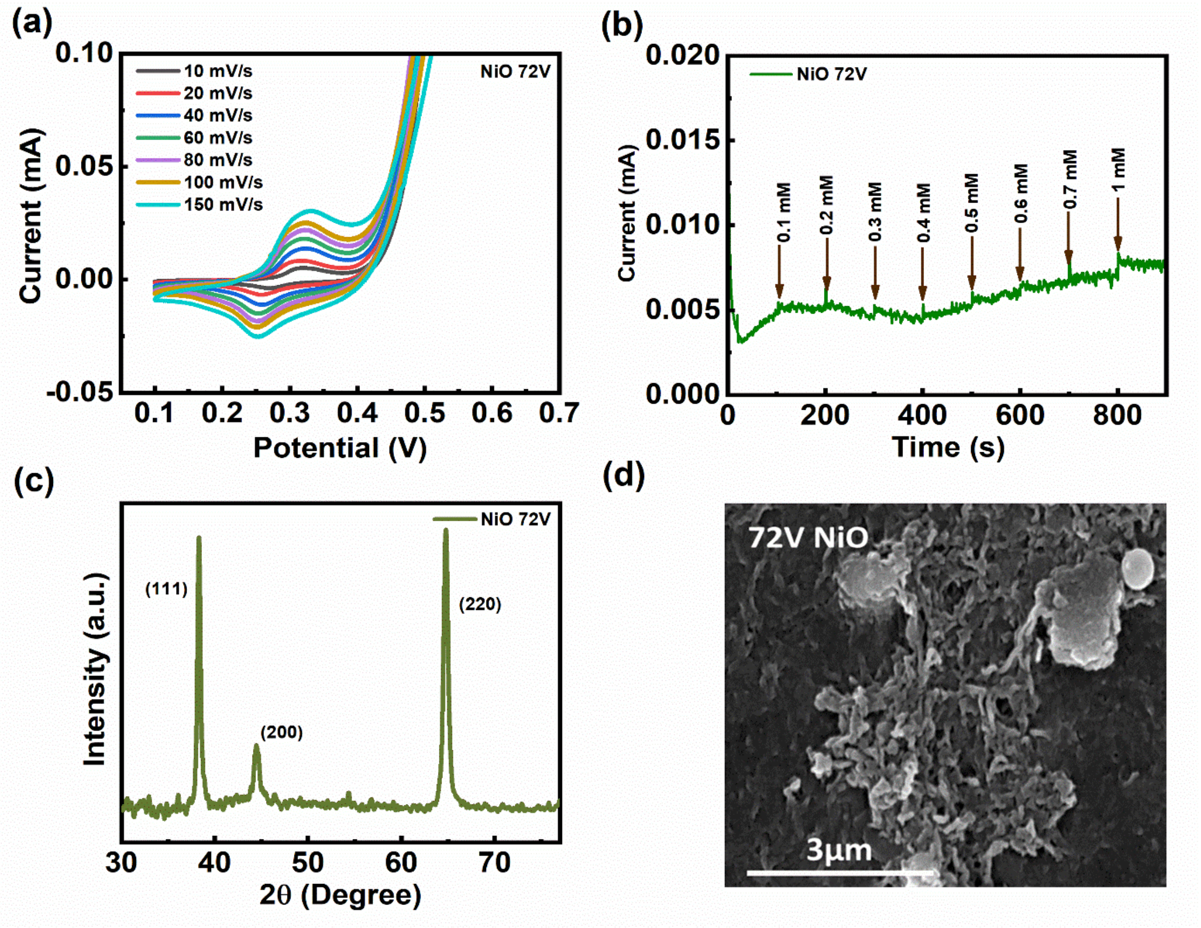

Figure S9. **(a)** Cyclic voltammetry (CV) curves of **NiO 72V** electrodes in a mixed solution of 0.5 mM glucose and 0.1 M NaOH at scan rates ranging from 10 to 150 mV/s. **(b)** Glucose sensing performance of amperometry (CA) measurements for **NiO 72V**. **(c)** X-ray diffraction (XRD) sample **NiO 72V**. **(d)** Scanning electron microscopy (SEM) images (top view) of sample **NiO 72V**.

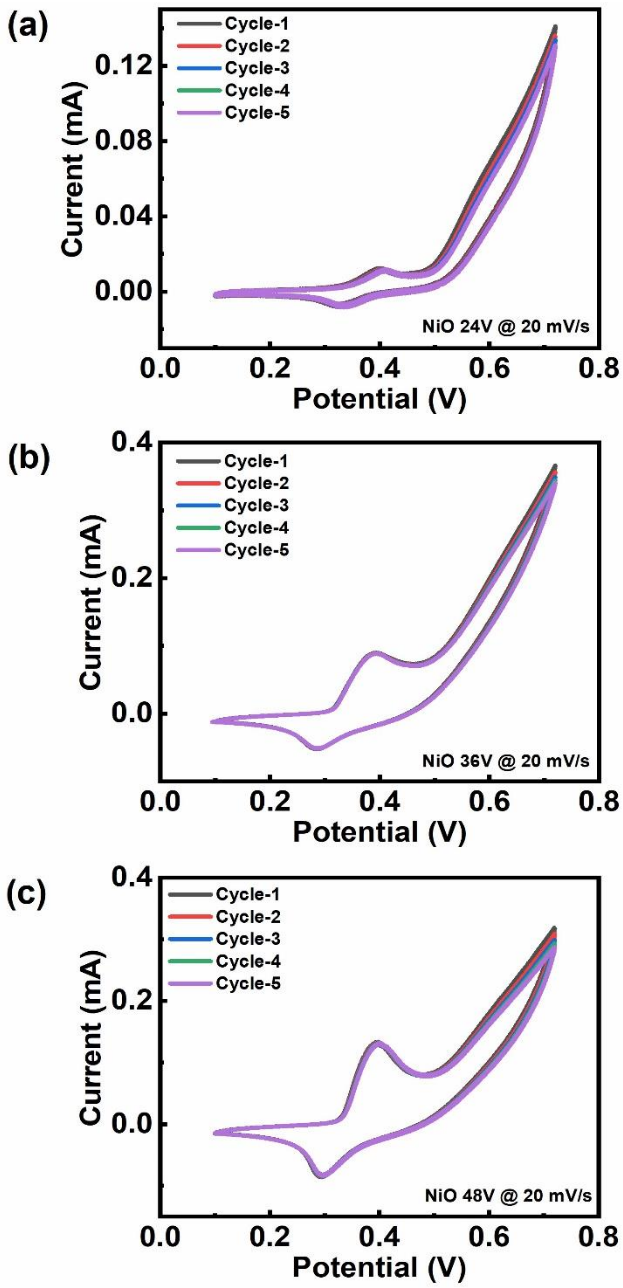

Figure S10. Multiple cycles cyclic voltammetry (CV) curves of **(a) NiO 24V**, **(b) NiO 36V**, and **(c) NiO 48V** electrodes at a scan rate of 20 mV/s.

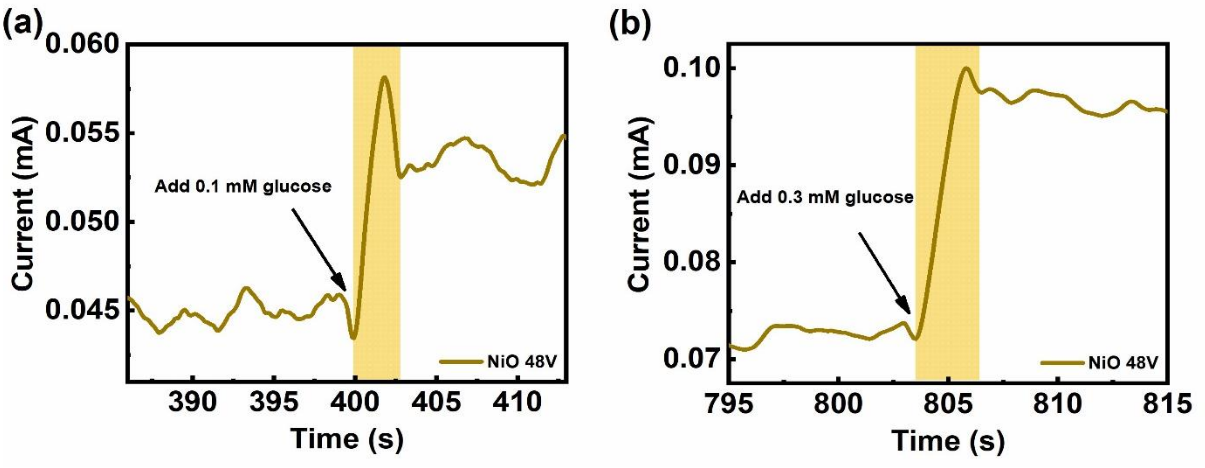

Figure S11. Response time with respect to the addition of **(a)** 0.1 mM glucose **(b)** 0.3 mM glucose.

Table S2. Comparisons of electrode sensitivities, limit of detection (LOD) values, and linear ranges of glucose sensors based on Ni and NiO composites published in related references.

**Electrode**	**Sensitivity (μA mM^{-1} cm^{-2})**	**LOD (μM)**	**Linear Range (mM)**	**Reference**
CuO/NiO-CT	586.7	0.04	0.0001-4.5	1
NiO_{1-x} Nanowires	661.0	6	0.05-17	2
$NiMoO_4$ NPs/CNF	301.8	0.05	0.0003-4.5	3
GOD-GA Ni/Cu-MOFs	26.1/11.0	0.51	0.001-20	4
Cu@Ni	420.0	62.5	0.2-12.2	5
NiSA-NC	799.4	0.22	0.0008-1.124	6
NiCo-LDH/MWCNTs	2.6	0.03	0.1-3000	7
CF-NiCo LDHN	53.9	0.03	0.1-0.7	8
Hierarchical Sheet-Like Ni-BDC/GCE	636	6.68	0.01–0.8	9
$NiFe_2O_4$-NiCo-LDH@rGO	111.9	12.94	0.035- 4.5	10
$CoMn_2O_4$@$Ni(OH)_2$	0.0065	0.26	0.0085- 1.8	11
Ni_3N NA	24.9	0.48	0.002-7.5	12
Ni/NiO/NC	76.0	0.20	0.0006-8.6	13
**NiO 24V**	265	1.00	0.1-1	This Work
**NiO 36V**	508	0.62	0.1-1	This Work
**NiO 48V**	**1202**	**0.25**	**0.1-1**	**This Work**

Table S3. Comparisons of electrode sensitivities, limit of detection (LOD) values, and linear ranges of enzyme-based glucose sensors published in related references.

**Electrode**	**Sensitivity (μA mM^{-1} cm^{-2})**	**LOD (μM)**	**Linear Range (mM)**	**Reference**
Ti_3C_2Tx /Gox	29.0	17.0	0.05 - 0.7	[14]
Gox/PAN-MWCNTs NFs/ PEDOT	92.9	2.3	-	[15]
FTO-CNTs/PEI/Gox	63.4	-	0.07 - 0.7	[16]
Fe_3O_4@PNE-Gox	97.3	6.1	0.2 - 24	[17]
Gox-rGO/Pt NPs@ZnMOF-74	64.5	1.8	0.006 - 6	[18]
Gox/Chitosan/GS/Prussian Blue	1.8	2.5	0.008 - 1	[19]

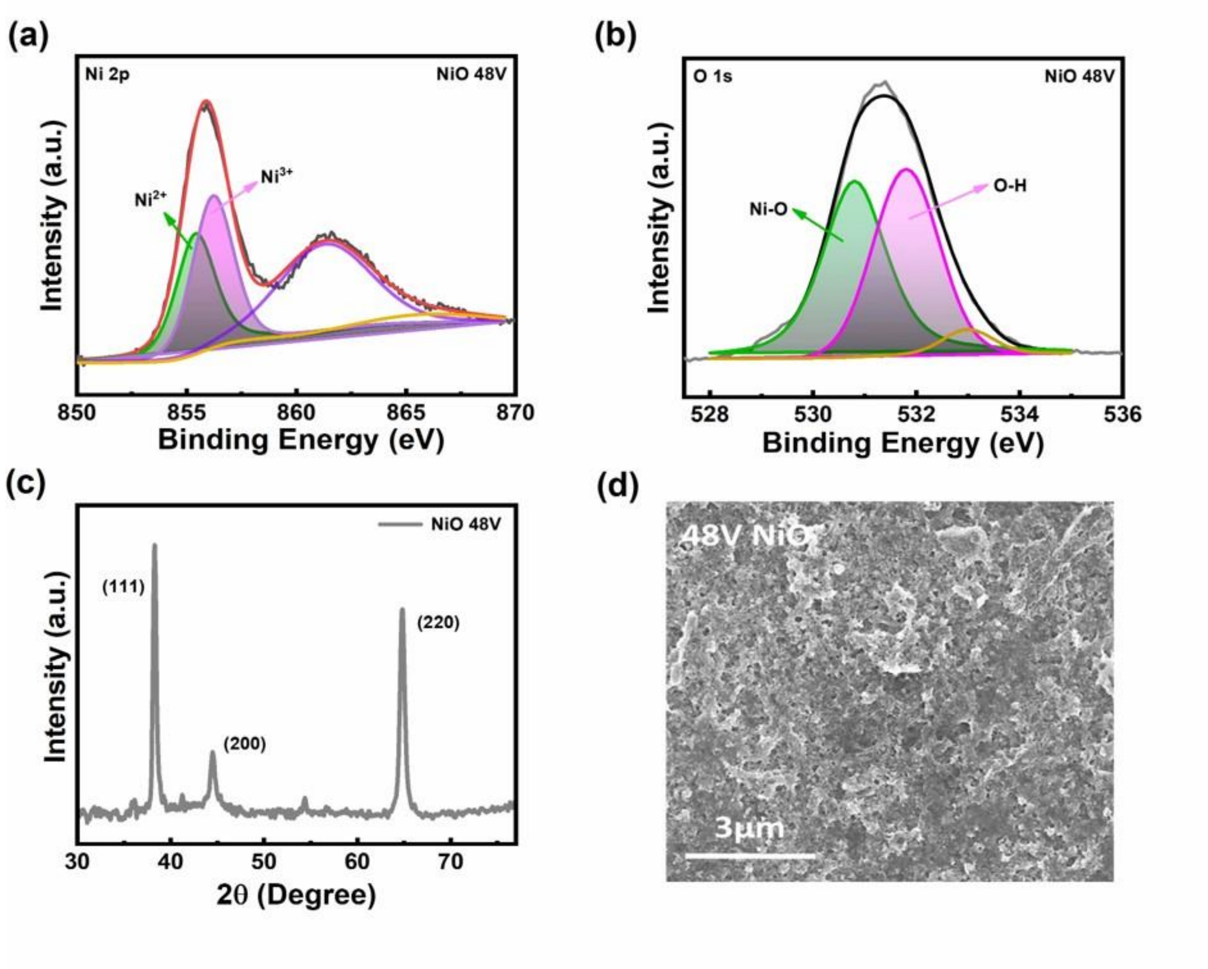

Figure S12. X-ray photoelectron spectroscopy (XPS) spectra of **(a)** Ni 2p and **(b)** O 1S for sample **NiO 48V**. **(c)** X-ray diffraction (XRD) patterns of **NiO 48V** sample. **(d)** Scanning electron microscopy (SEM) images (top view) of sample **NiO 48V** after 10 days of consecutive measurements.

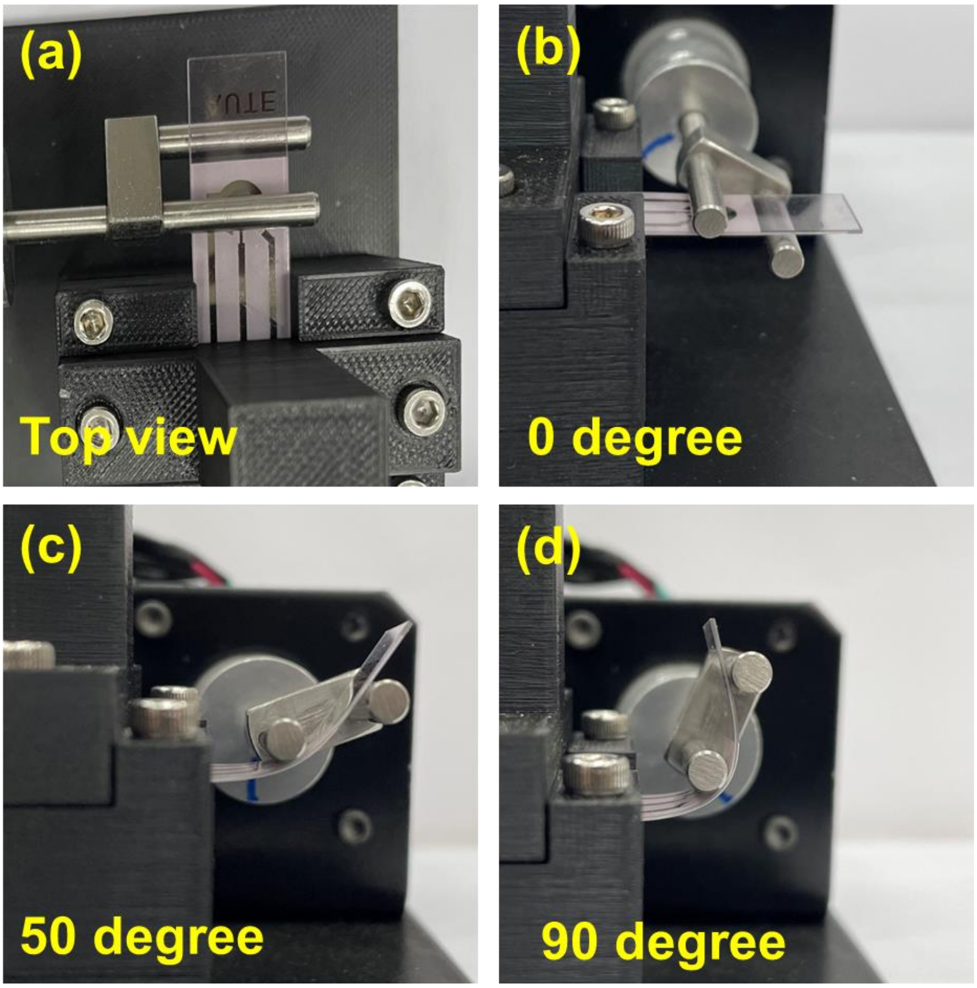

Figure S13. Different bending angle images of **NiO 48V** electrode taken by **(a)** top view (without bending) and side views with **(b)** 0 degree, **(c)** 50 degree, and **(d)** 90 degree bendings.

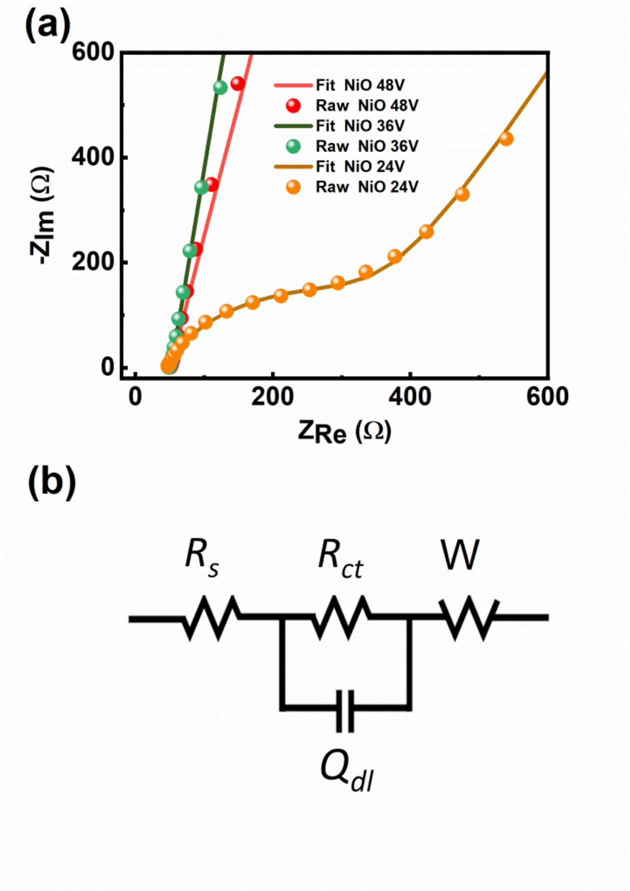

Figure S14. Electrochemical impedance spectroscopy (EIS) of **(a) NiO 24V**, **NiO 36V**, and **NiO 48V** electrodes in 0.1 M NaOH. **(b)** An equivalent circuit model used to fit the EIS data of the NiO electrodes.

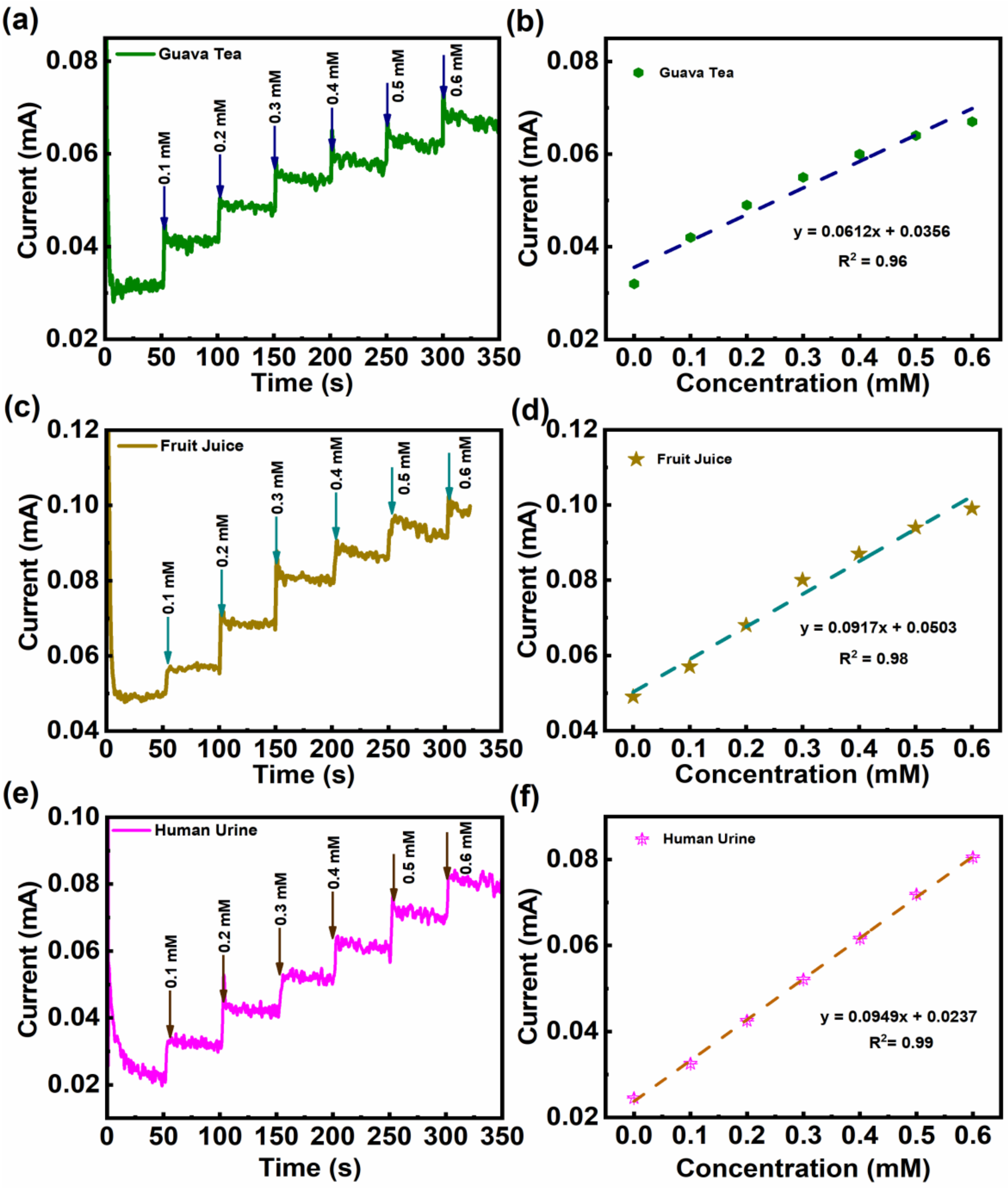

Figure S15. (a) Amperometry (CA) measurements of **NiO 48V** electrode with sequential additions of 0.1 mM glucose in 0.1 M NaOH solution containing guava tea **(c)** fruit juice, and **(e)** human urine at 0.45 V. Calibration curves of **(b)** guava tea, **(d)** fruit juice, and **(f)** human urine where the dashed line gave the best linear least-square fitting for the correlation between the current and glucose concentration.

REFERENCES

(1) Archana, V.; Xia, Y.; Fang, R.; Gnana Kumar, G. Hierarchical CuO/NiO-Carbon Nanocomposite Derived from Metal Organic Framework on Cello Tape for the Flexible and High Performance Nonenzymatic Electrochemical Glucose Sensors. *ACS Sustainable Chemistry & Engineering* **2019**, *7* (7), 6707-6719. DOI: 10.1021/Acssuschemeng.8b05980.
(2) Wang, L.; Lu, W.; Zhu, W.; Wu, H.; Wang, F.; Xu, X. A Photoelectrochemical Sensor for Highly Sensitive Detection of Glucose Based on Au–NiO1–X Hybrid Nanowires. *Sensors and Actuators B: Chemical* **2020**, *304*, 127330. DOI: Https://Doi.Org/10.1016/J.Snb.2019.127330.
(3) Rani, S. D.; Ramachandran, R.; Sheet, S.; Aziz, M. A.; Lee, Y. S.; Al-Sehemi, A. G.; Pannipara, M.; Xia, Y.; Tsai, S.-Y.; Ng, F.-L. NiMoO4 Nanoparticles Decorated Carbon Nanofiber Membranes for the Flexible and High Performance Glucose Sensors. *Sensors and Actuators B: Chemical* **2020**, *312*, 127886. DOI: https://doi.org/10.1016/j.snb.2020.127886.
(4) Wang, B.; Luo, Y.; Gao, L.; Liu, B.; Duan, G. High-Performance Field-Effect Transistor Glucose Biosensors Based on Bimetallic Ni/Cu Metal-Organic Frameworks. *Biosensors and Bioelectronics* **2021**, *171*, 112736. DOI: Https://Doi.Org/10.1016/J.Bios.2020.112736.
(5) Ghosh, R.; Li, X.; Yates, M. Z. Nonenzymatic Glucose Sensor Using Bimetallic Catalysts. *ACS Applied Materials & Interfaces* **2024**, *16* (1), 17-29. DOI: 10.1021/acsami.3c10167.
(6) Yin, H.; Bai, X.; Yang, Z. Activating Ni Nanoparticles into Ni Single Atoms by N Doping for High-Performance Electrochemical Sensing of Glucose. *Chemical Engineering Journal* **2023**, *478*, 147510. DOI: Https://Doi.Org/10.1016/J.Cej.2023.147510.
(7) Zhu, Y.; Qian, J.; Xu, K.; Ouyang, W.; Yang, J.; Yang, N. Hollow Nanocages Heterostructured NiCo-LDH/MWCNTs Electrocatalyst for Highly Sensitive and Non-Invasive Detection of Saliva Glucose. *Chemical Engineering Journal* **2024**, *485*, 149795. DOI: Https://Doi.Org/10.1016/J.Cej.2024.149795.
(8) Lo, C.-T.; Wu, Y.-S.; Huang, S.-M.; Tsai, P.-J.; Lee, C.-L. Carbon Fibre-Supported Hierarchical NiCo Layered Double Hydroxide Nanosheets as Non-Enzymatic Glucose Sensors for Sport Drinks and Serum. *Food Chemistry* **2022**, *383*, 132383. DOI: https://doi.org/10.1016/j.foodchem.2022.132383.
(9) Gumilar, G.; Kaneti, Y. V.; Henzie, J.; Chatterjee, S.; Na, J.; Yuliarto, B.; Nugraha, N.; Patah, A.; Bhaumik, A.; Yamauchi, Y. General Synthesis of Hierarchical Sheet/Plate-Like M-BDC (M= Cu, Mn, Ni, And Zr) Metal–Organic Frameworks for Electrochemical Non-Enzymatic Glucose Sensing. *Chemical Science* **2020**, *11* (14), 3644-3655. DOI: 10.1039/C9SC05636J.
(10) Chu, D.; Li, F.; Song, X.; Ma, H.; Tan, L.; Pang, H.; Wang, X.; Guo, D.; Xiao, B. A Novel Dual-Tasking Hollow Cube NiFe2O4-NiCo-LDH@rGO Hierarchical Material for High Preformance Supercapacitor and Glucose Sensor. *Journal of Colloid and Interface Science* **2020**, *568*, 130-138. DOI: https://doi.org/10.1016/j.jcis.2020.02.012.
(11) Wang, X.; Hao, L.; Du, R.; Wang, H.; Dong, J.; Zhang, Y. Synthesis of Unique Three-Dimensional CoMn2O4@Ni(OH)2 Nanocages via Kirkendall Effect for Non-Enzymatic Glucose Sensing. *Journal of Colloid and Interface Science* **2024**, *653*, 730-740. DOI: Https://Doi.Org/10.1016/J.Jcis.2023.09.098.
(12) Luo, J.; Zhao, D.; Yang, M.; Qu, F. Porous Ni3N Nanosheet Array as a Catalyst for Nonenzymatic Amperometric Determination of Glucose. *Microchimica Acta* **2018**, *185* (4), 229. DOI: 10.1007/s00604-018-2764-z.

(13) Liang, H.; Luo, Y.; Xiao, Y.; Chen, R.; Wang, L.; Song, Y. Ni/NiO/Carbon Derived from Covalent Organic Frameworks for Enzymatic-Free Electrochemical Glucose Sensor. *Ceramics International* **2024**, *50* (1, Part A), 977-984. DOI: https://doi.org/10.1016/j.ceramint.2023.10.188.
(14) Myndrul, V.; Coy, E.; Babayevska, N.; Zahorodna, V.; Balitskyi, V.; Baginskiy, I.; Gogotsi, O.; Bechelany, M.; Giardi, M. T.; Iatsunskyi, I. Mxene Nanoflakes Decorating ZnO Tetrapods for Enhanced Performance of Skin-Attachable Stretchable Enzymatic Electrochemical Glucose Sensor. *Biosensors and Bioelectronics* **2022**, *207*, 114141.
(15) Çetin, M. Z.; Guven, N.; Apetrei, R.-M.; Camurlu, P. Highly Sensitive Detection of Glucose via Glucose Oxidase Immobilization onto Conducting Polymer-Coated Composite Polyacrylonitrile Nanofibers. *Enzyme and Microbial Technology* **2023**, *164*, 110178.
(16) Lin, M.-H.; Gupta, S.; Chang, C.; Lee, C.-Y.; Tai, N.-H. Carbon Nanotubes/Polyethylenimine/Glucose Oxidase as a Non-Invasive Electrochemical Biosensor Performs High Sensitivity for Detecting Glucose in Saliva. *Microchemical Journal* **2022**, *180*, 107547.
(17) Jędrzak, A.; Kuznowicz, M.; Rębiś, T.; Jesionowski, T. Portable Glucose Biosensor Based on Polynorepinephrine@ Magnetite Nanomaterial Integrated with a Smartphone Analyzer for Point-of-Care Application. *Bioelectrochemistry* **2022**, *145*, 108071.
(18) Uzak, D.; Atiroğlu, A.; Atiroğlu, V.; Çakıroğlu, B.; Özacar, M. Reduced Graphene Oxide/Pt Nanoparticles/Zn-MOF-74 Nanomaterial for a Glucose Biosensor Construction. *Electroanalysis* **2020**, *32* (3), 510-519.
(19) Li, B.; Wu, X.; Shi, C.; Dai, Y.; Zhang, J.; Liu, W.; Wu, C.; Zhang, Y.; Huang, X.; Zeng, W. Flexible Enzymatic Biosensor Based on Graphene Sponge for Glucose Detection in Human Sweat. *Surfaces and Interfaces* **2023**, *36*, 102525.
